# Potent ferroptosis agent RSL3 induces cleavage of Pyroptosis-Specific gasdermins in Cancer cells

**DOI:** 10.1038/s41598-025-11368-1

**Published:** 2025-07-12

**Authors:** William G. Herrick, Huong-Lan Tran, Francesca R. Tomaino, Brittany Beall, Jeevan Govindharajulu, Dominic Esposito, Laura Kuhlmann, Ralph E. Parchment, James H. Doroshow, Apurva K. Srivastava

**Affiliations:** 1 Clinical Pharmacodynamics Biomarker Program, Frederick National Laboratory for Cancer Research, Leidos Biomedical Research, Inc., Frederick, MD 21702 USA; 2 Protein Expression Laboratory, Cancer Research Technology Program, Frederick National Laboratory for Cancer Research, Leidos Biomedical Research, Inc., Frederick, MD 21702 USA; 3 Division of Cancer Treatment and Diagnosis, National Cancer Institute, Bethesda, MD 20892 USA; 4 Center for Cancer Research, National Cancer Institute, Bethesda, MD 20892 USA

**Keywords:** Ferroptosis, Pyroptosis, Cancer, RSL3, Gasdermin, Cytokine, Cancer, Cytokines

## Abstract

Ferroptosis is a form of iron-dependent cell death of interest for the development of novel anti-cancer therapies. Ferroptosis research uses a process of elimination based on assumed ferroptosis-specific inducers and inhibitors; these molecules however have off-target effects and cannot provide a comprehensive picture of overlapping pathways. We investigated whether pyroptosis—a form of inflammatory cell death—is initiated in cancer cells following treatment with the ferroptosis inducer RSL3. We treated 6 cancer cell lines with RSL3 alone or in combination with inhibitors of ferroptosis (Ferrostatin-1), caspases (zVADfmk), necroptosis (Necrostatin-1), BID (BI-6C9), or STING (H-151). Biomarkers of pyroptosis and ferroptosis were assessed using our novel quantitative multiplex immunoassay. Increased secretion of pyroptosis-associated cytokines (IL-1α, IL-1β, IL-18), and gasdermin D and E (GSDMD/E) cleavage with parallel loss of respective full-length proteins—both hallmarks of pyroptosis—were recorded in 5/6 cell lines following RSL3 treatment. RSL3 cytotoxicity was blocked by Ferostatin-1; BID and STING inhibitors also prevented GSDMD/E cleavage. We conclude that the ferroptosis-inducer RSL3 triggers pyroptosis in cancer cells; further work is required to elucidate the role of mitochondria in this process. Measurement of pathway-specific protein biomarkers is therefore necessary to identify the exact mechanism of action of novel cytotoxic agents.

**Supplementary Information:**

The online version contains supplementary material available at 10.1038/s41598-025-11368-1.

## Introduction

Ferroptosis is a caspase-independent form of cell death driven by iron-catalyzed phospholipid peroxidation^1^; it is regulated by 2 major antioxidant systems: glutathione peroxidase 4 (GPx4), which reduces phospholipid peroxides in a glutathione-dependent reaction^2,3^; and the ferroptosis suppressor protein (FSP1) that catalyzes the regeneration of Coenzyme Q10^3,4^. Historically, cell death was classified largely by morphological features, but this approach is insufficient given that at least 3 different forms of cell death (ferroptosis, pyroptosis, and necroptosis) induce the so-called ‘type III’ (necrotic) morphology^5^. Translational research has lagged in the development of definitive ferroptosis biomarkers^6^. Phospholipid hydroperoxides are predicted to be the most reliable biomarker for ferroptosis, but measurement by LC-MS is technically challenging^7^. Increased membrane expression of transferrin receptor 1 (TfR), which facilitates cellular iron import, has also been shown to correlate with ferroptosis^8^. The 2 most commonly used ferroptosis inducers are RSL3, a GPx4 inhibitor^9^, and erastin, which inhibits cystine uptake by system xC^−^ causing glutathione depletion^10^. Ferroptosis is presumed if cell death is inhibited by both lipophilic antioxidants (e.g. ferrostatin-1) and iron chelators but not by caspase or Receptor Interacting Protein Kinases (RIPK) inhibitors^11,12^. Employing a process of elimination dependent on pathway inhibitors is confounded by dose-dependent off-target effects of these compounds^13,14^, which may explain why different researchers draw distinct conclusions about the induced cell death pathway of a given compound^15,16^.

Pyroptosis is a form of inflammatory cell death accompanied by release of pro-inflammatory cytokines such as interleukin-1β (IL-1β) and interleukin-18 (IL-18)^17^. Pyroptosis is executed when gasdermin proteins are cleaved by proteases (primarily, but not exclusively caspases) into N- and C–terminal domains, followed by oligomerization of the N-terminal fragments into membrane pores leading to cell swelling and lysis^18–21^. Mechanistically, pyroptosis has been shown to overlap with other forms of cell death such as apoptosis and necroptosis, where caspases play important roles^22–24^, but no co-occurrences have been reported to date with ferroptosis. Pyroptosis and ferroptosis have certain features in common including plasma membrane permeabilization with cell swelling^25,26^, cyclic GMP-AMP synthase (cGAS)–stimulator of interferon genes (STING) signaling^27,28^, BH3-interacting domain death agonist (BID) activation^29,30^, and oxidative stress^31,32^. Lipid peroxidation has also been linked to GSDMD oligomerization and membrane binding, inflammasome activation, and caspase-1/4/5/11 activation^33–35^; however gasdermin or pro-inflammatory cytokine activation has not been reported to date in ferroptosis^36–38^.

In this study we investigated whether the ferroptosis inducer RSL3 could initiate other forms of cell death. RSL3-triggered pyroptosis is evidenced by cleaved GSDMD and/or GSDME detected by our sensitive sandwich immunoassays^39^ and release of pyroptosis-associated cytokines (IL-1α, IL-1β and IL-18). We discuss the translational research implications of this observation that a specific ferroptosis inducer also triggers pyroptosis.

## Materials & methods

### Cell culture

All cell lines were cultured using RPMI-1640 (ATCC modification; Gibco) with 10% FBS (Rockland) in a 37 °C incubator with 5% CO_2_. BxPC3, SU-DHL-5 and HT-29 cells were purchased from ATCC; MDA-MB-231, NCI-H522, ACHN, UO-31, and THP-1 cells were obtained from the NCI Biological Testing Branch (Frederick, MD). See https://dctd.cancer.gov/drug-discovery-development/reagents-materials/animal-tumor-models/dctd-tumor-repository-catalog for details on quality control and cell line characterization. The HAP1 GSDMD knockout cell line was obtained from Horizon Discovery (Cambridge, UK).

For adherent cell lines, biomarker experiments were performed using T-175 tissue culture–treated flasks (Corning) (Supplemental Table S1). Approximately 2 days after seeding, the growth media was replaced with media containing RSL3 and 5% FBS (final DMSO concentration 0.1%). In inhibitor combination experiments, cells were pre-treated with inhibitors (Supplemental Table S2) for 1 h before the growth media was replaced with media containing both RSL3 and inhibitor. SU-DHL-5 suspension cells were treated at 0.25 × 10^6^ cells/mL in media with 10% FBS and the 1 h inhibitor pre-treatment was not performed to minimize cell handling. Cell type-specific seeding densities and compound concentrations are outlined in Supplemental Table S1; drug and inhibitor concentrations are shown in Supplemental Table S2. RSL3 concentrations were selected to reliably induce a strong cytotoxic effect after 24 h.

After approximately 24 h of treatment, cells were collected by scraping into media on ice, washed twice by resuspension in ice-cold DPBS, and pelleted at 500 x g, 4 °C for 5 min, and then washed a final time with ice-cold DPBS containing protease and phosphatase inhibitors (Roche). Cells were pelleted at 10,000 x g, 4 °C for 5 min. DPBS was aspirated, and cell pellets were frozen on dry ice, and then stored at −80 °C until protein extraction.

THP-1 cells were used as a positive control of pyroptosis^40^; for this purpose 1 × 10^6^ cells/mL were differentiated into adherent cells with 50 ng/mL phorbol 12-myristate 13-acetate (PMA; Sigma Aldrich) for 3 days, then primed with 10 µg/mL lipopolysaccharide (LPS) from *E. coli* (Sigma Aldrich) overnight. The next day, cells were treated with vehicle (0.1% ethanol) or 20 µM nigericin (Cayman Chemicals) for 30 min. Samples were collected as described above.

HT-29 cells were used as a positive control for necroptosis and MLKL phosphorylation; 1.7 × 10^6^ cells were seeded in T-175 flasks the day before treatment with 10 µM emricasan for 30 min to inhibit caspases followed by addition of 20 ng/mL TNFα and 1 µM tolinapant. Cell pellets were collected after 6 h of treatment.

### Total protein extraction

Whole cell lysates were generated from cell pellets as previously reported^41^. Briefly, cold Cell Extraction Buffer (Invitrogen) containing protease and phosphatase inhibitors (Roche) was added to cell pellets on ice; pellets were resuspended by pipetting and vortexing, and then incubated on ice for 1 h with shaking and vortexed every 20 min. Samples were centrifuged at 13,000 x g, 4 °C for 5 min, and supernatants were collected and stored at −80 °C. Protein concentrations were determined using the Bradford method (Bio-Rad).

### ATP measurement in cell cultures

Cells were seeded in 96-well white-walled CELLSTAR plates (Greiner Bio-One) in a total volume of 0.1 mL/well and treated as described above (see Supplemental Table S1 for seeding densities). Inhibitors were added at the same time as RSL3 to minimize handling, without pre-treatment. Relative quantities of ATP were assayed as a measure of cell viability using CellTiter Glo-2.0 (Promega) according to the manufacturer’s instructions, and luminescence was read with a Tecan Infinite M1000 multimode plate reader.

### Cytokine measurement in cell culture supernatants

Cells were seeded in 12- or 24–well plates (Corning) to a final volume of 1 mL/well and 0.5 mL/well respectively, and treated with RSL3 for 4–24 h as described above. After removing cells and debris by centrifugation (800 x g, 4 °C for 10 min), supernatants were frozen on dry ice and stored at −80 °C. Supernatants were concentrated approximately 3-6-fold using Amicon Ultra devices with a 10 kDa nominal molecular weight limit (EMD Millipore) according to the manufacturer’s instructions and sample concentration factors determined for each sample (starting sample volume ÷ eluted volume). Cytokines secreted in concentrated cell culture supernatants were assayed with a customized MILLIPLEX MAP Human Cytokine panel A (EMD Millipore) according to the manufacturer’s instructions.

### Ferroptosis and pyroptosis multiplex assay

Assay details are provided in the Supplemental Methods.

### Statistical analysis

Multiplex biomarker data were generated using Luminex xPONENT software. Relative transferrin receptor quantitation was performed using blank-subtracted assay signals. For the remaining targets, analyte concentrations in samples were interpolated from calibrator standard curves fitted to 5-parameter logistic regressions (Bio-Plex Manager v6.1 or newer). Analyte concentrations were normalized to total protein load (pg analyte/µg total protein), and then to the experiment-matched vehicle sample average (expressed as % of vehicle). Every biomarker data point represents the vehicle-normalized value obtained from 1 sample (flask), and each treatment was performed on a minimum of 3 different days with 2 flasks per treatment per experiment, unless otherwise specified. Cell viability data was normalized to the vehicle group average for each experiment with 3 technical replicates per group and 3 independent experiments; each data point represents the replicate-averaged value obtained from 1 experiment. Similarly, secreted cytokine data from 2 to 3 independent experiments for each cell line were divided by sample concentration factors and then normalized to vehicle group averages. Null hypothesis testing of treatment compared to vehicle control was performed with 1-sample t-tests using µ = 100% (representing no change from vehicle) and a cutoff significance level of *α* = 0.05. The effect of inhibitors on RSL3-induced changes in ATP or biomarker levels was evaluated by one-way ANOVA with correction for multiple comparisons and each biomarker was considered independently. All statistical analyses were performed in GraphPad Prism v.10. Probability values are indicated in the figures (* *p* < 0.05; ** *p* < 0.01; *** *p* < 0.001; **** *p* < 0.0001).

## Results

### RSL3 induces Fer-1–preventable necrotic morphologies and ATP depletion that are not blocked by caspase and necroptosis inhibitors

**Fig. 1:**
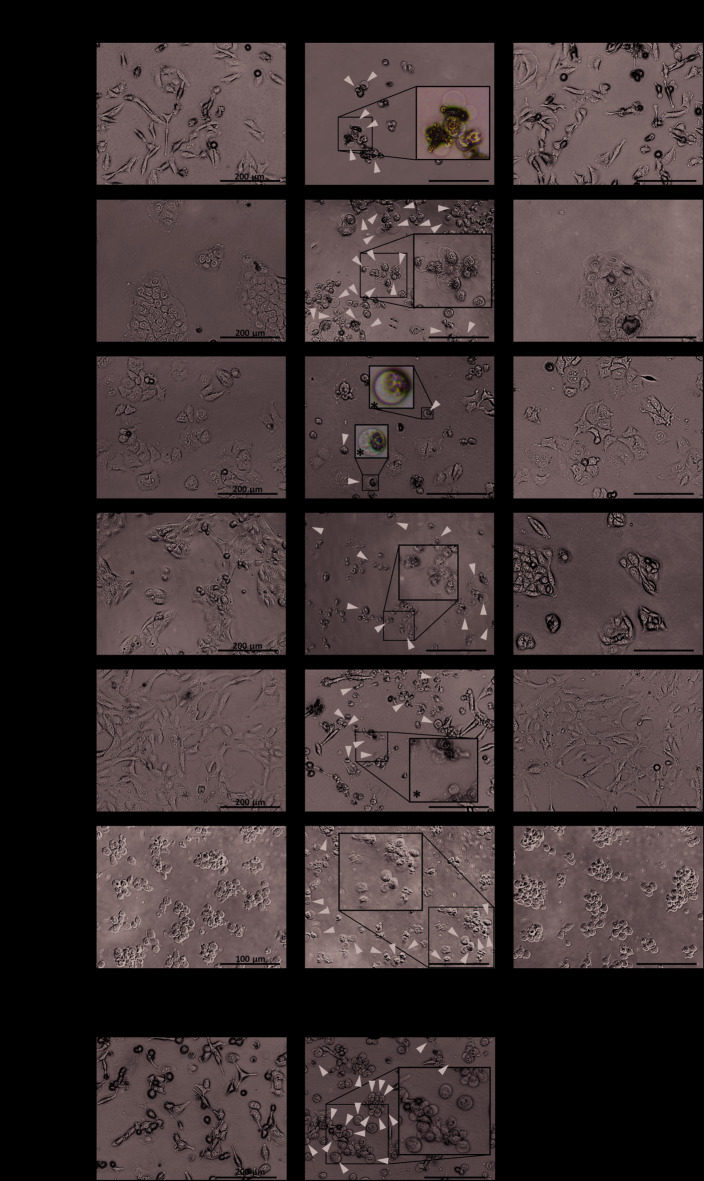
RSL3 Induces Necrotic Morphologies in Cancer Cell Lines. (A) RSL3 induces necrotic morphology in 6 cancer cell lines which is prevented by the lipophilic antioxidant and ferroptosis inhibitor Fer-1. This morphology is characterized by a swollen, rounded appearance with cellular organelles compressed to one side. Scale bars for SU-DHL5: 100 μm; all other scale bars: 200 μm. (B) THP-1 macrophages primed with LPS and treated with 20 µM nigericin for 30 min to induce pyroptosis display the same basic morphological features as RSL3-treated cells.

**Fig. 2:**
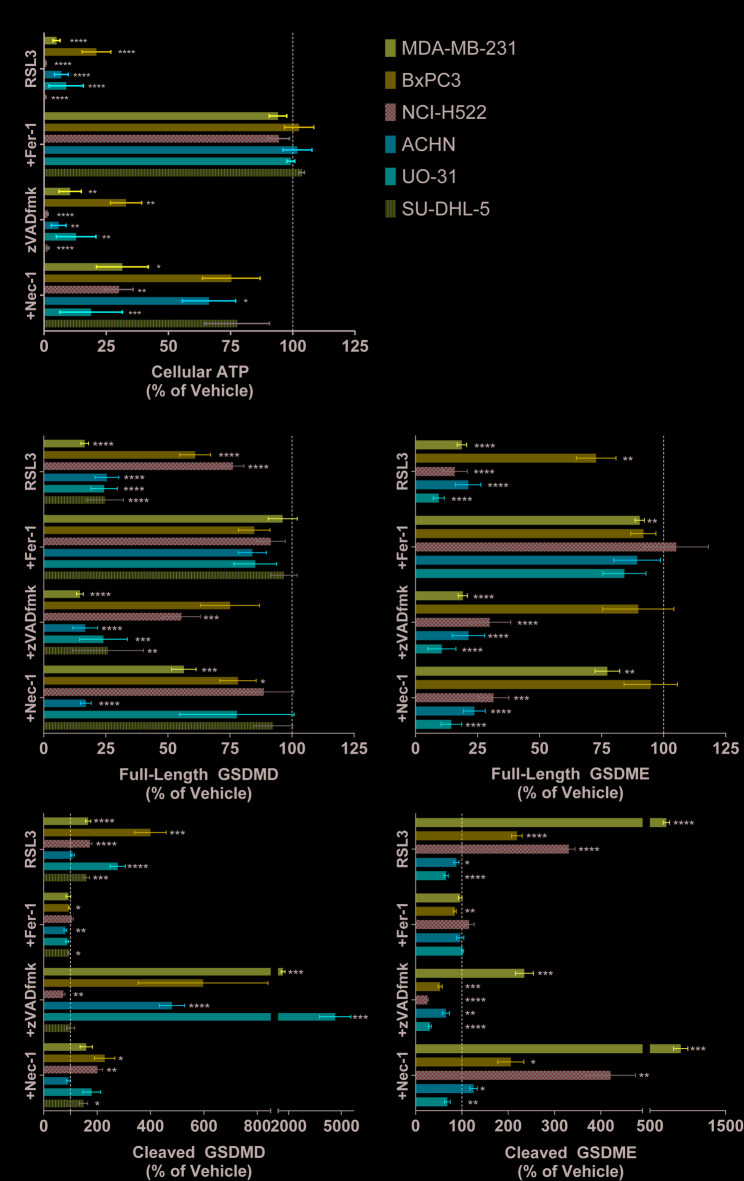
RSL3 Induces Gasdermin Cleavage in Cancer Cells and Pyroptosis is Partially Prevented by Fer-1 and Nec-1, but Not by Caspase Inhibition. (A) ATP levels relative to vehicle control following 1 day of RSL3 treatment with or without ferroptosis inhibitor Fer-1, pan-caspase inhibitor (zVADfmk), or RIPK1 and IDO1/2 inhibitor (Nec-1) co-treatment. All *N* ≥ 3. Error bars: SEM. Probability values: * *p* < 0.05; ** *p* < 0.01; *** *p* < 0.001; **** *p* < 0.0001. (B) Full-length and cleaved GSDMD and GSDME levels relative to vehicle control following 1 day of RSL3 treatment with or without ferroptosis inhibitor Fer-1, pan-caspase inhibitor (zVADfmk), or RIPK1 and IDO1/2 inhibitor (Nec-1) co-treatment. All *N* ≥ 3. Error bars: SEM. Probability values: * *p* < 0.05; ** *p* < 0.01; *** *p* < 0.001; **** *p* < 0.0001.

The morphologies, ATP content (as a surrogate for cell viability), and biomarkers of pyroptosis and ferroptosis were evaluated in 6 cancer cell lines treated with the ferroptosis inducer RSL3, alone or in combination with the ferroptosis inhibitor Fer-1, for 24 h (Supplemental Tables S1, S2). When treated with RSL3, each cell line exhibited necrotic morphologies observable by brightfield microscopy (Fig. 1A), which were prevented by Fer-1. These features include cell rounding and swelling into a large bubble of thinned plasma membrane with organelles compacted to one side (Fig. 1A). The appearance is indistinguishable from the morphology seen in the commonly used model of pyroptosis in which THP-1 macrophages are primed with LPS and treated with nigericin^40^ (Fig. 1B). Intracellular ATP content was reduced by > 75% in all 6 cell lines following RSL3 treatment, and the effect was completely prevented by Fer-1 (Fig. 2A). Using our custom immunoassay^39^ we evaluated GPx4 and full-length TfR levels following RSL3 and inhibitor treatment as putative adjacent markers of ferroptosis. As expected, RSL3 reduced GPx4 protein levels in all cell lines, except for NCI-H522 (Supplemental Fig. S1), in which GPx4 levels increased by 13% (*p* = 0.026). Levels of full-length TfR decreased in 3 cell lines and increased in 2 cell lines (Supplemental Fig. S1) following RSL3 treatment. Co-treatment with Fer-1 counteracted loss of GPx4 only in SU-DHL-5 and UO-31 cells and increased GPx4 in NCI-H522 cells to 46% over vehicle. Fer-1 treatment prevented TfR modulation by RSL3 in all cell lines (Supplemental Fig. S1).

To investigate contributions from other cell death pathways, we also evaluated cellular morphologies, biomarkers, and ATP content in cells treated with RSL3 in combination with a pan-caspase inhibitor (zVADfmk), or a necroptosis inhibitor (Necrostatin-1, Nec-1). Nec-1 partially rescued 5/6 cell lines from RSL3-induced ATP depletion, whereas caspase inhibitor zVADfmk did not prevent RSL3-induced ATP depletion (Fig. 2A). Neither inhibitor reduced the prevalence of cells with necrotic morphologies (Supplemental Fig. S2). Co-treatment with zVADfmk did not substantially affect GPx4 or TfR levels compared to RSL3 treatment alone (Supplemental Fig. S1). Nec-1 co-treatment followed a pattern similar to that observed following Fer-1 co-administration (Supplemental Fig. S1). Although ferroptosis does not require caspase activity, we detected an increase in active caspase-3 levels in 2/6 cell lines following RSL3 treatment (Supplemental Fig. S1), which was completely blocked by Fer-1 and partially prevented by zVADfmk.

### RSL3 treatment induces gasdermin cleavage, which is partially affected by caspase inhibitors

Using our custom multiplex immunoassay^39^ we detected significant (*p* < 0.001) RSL3-induced cleavage of GSDMD in 5/6 cell lines and of GSDME in 3/5 cell lines, accompanied by a reduction in full-length GSDMD and GSDME (Fig. 2B). Fer-1 co-treatment inhibited the effects of RSL3 on cleaved and full-length GSDMD/E levels. SU-DHL-5 cells do not express GSDME and are not included in GSDME-related plots (Supplemental Fig. S3A). GSDMD protein is not detectable in NCI-H522 cells by Western blot, although low amounts could be detected by our assay (Supplemental Fig. S3B). See Supplemental Materials for complete WB pictures. Only the ACHN cell line did not have significant induction of either GSDMD or GSDME cleavage following RSL3 treatment, although the full-length respective protein levels decreased significantly (*p* < 0.0001).

Caspase inhibitor treatment had various effects on the cell lines, mostly inhibiting GSDME cleavage in 4/5 cell lines relative to RSL3 treatment (Fig. 2B; *p* < 0.05). Paradoxically, zVADfmk increased GSDMD cleavage in 3 cell lines (Fig. 2B; *p* < 0.05), including the ACHN cell line where no significant GSDMD cleavage was detected after treatment with RSL3 alone. In the NCI-H522 and SU-DHL-5 cell lines, caspase inhibition suppressed RSL3-induced GSDMD cleavage to vehicle levels or lower (Fig. 2B). Nec-1 co-treatment did not markedly affect GSDMD/E cleavage compared to RSL3 single-agent treatment, but it significantly inhibited loss of full-length GSDMD in 3/6 cell lines tested (*p* < 0.05), and prevented loss of full-length GSDME in MDA-MB-231 cells (Fig. 2B).

Western blot assays confirmed that RSL3 induced GSDME cleavage in BxPC3, NCI-H522, and MDA-MB-231 cell lines, which closely matched the results seen with our Luminex assay (Supplemental Figure S4; see also Supplemental Materials for complete WB pictures). However, we were unable to detect cleaved GSDMD in RSL3-treated cancer cells by Western blot, while a positive control (LPS-primed THP-1 macrophages treated with nigericin) showed the predicted cleaved GSDMD bands (Supplemental Fig. S5A). We established lower limits of detection (LODs) for GSDMD-FL and clGSDMD in our assay using HAP1 GSDMD knockout (KO) cell lysate to confirm that GSDMD cleavage levels are not due to noise or non-specific binding (Supplemental Fig. S5B). See Supplemental Materials for complete WB pictures. To further rule-out non-specific contributions to the cleaved GSDMD Luminex assay signal, we performed additional validations including cleaved GSDMD peptide competition with antigen peptide and removal of the GSDMD detection antibody from the multiplex. Cleaved GSDMD Luminex assay signal was reduced by an average of 100% with peptide competition (range: 97 − 107%) and by an average of 76.5% when removing GSDMD detection antibody (range: 40–99%, 7/8 samples > 70%), thus confirming that cleaved GSDMD signals in the RSL3-treated samples are not due to non-specific binding (Supplemental Fig. S5B).

### RSL3-induced cytotoxicity and gasdermin cleavage is prevented by STING or BID Inhibition

**Fig. 3:**
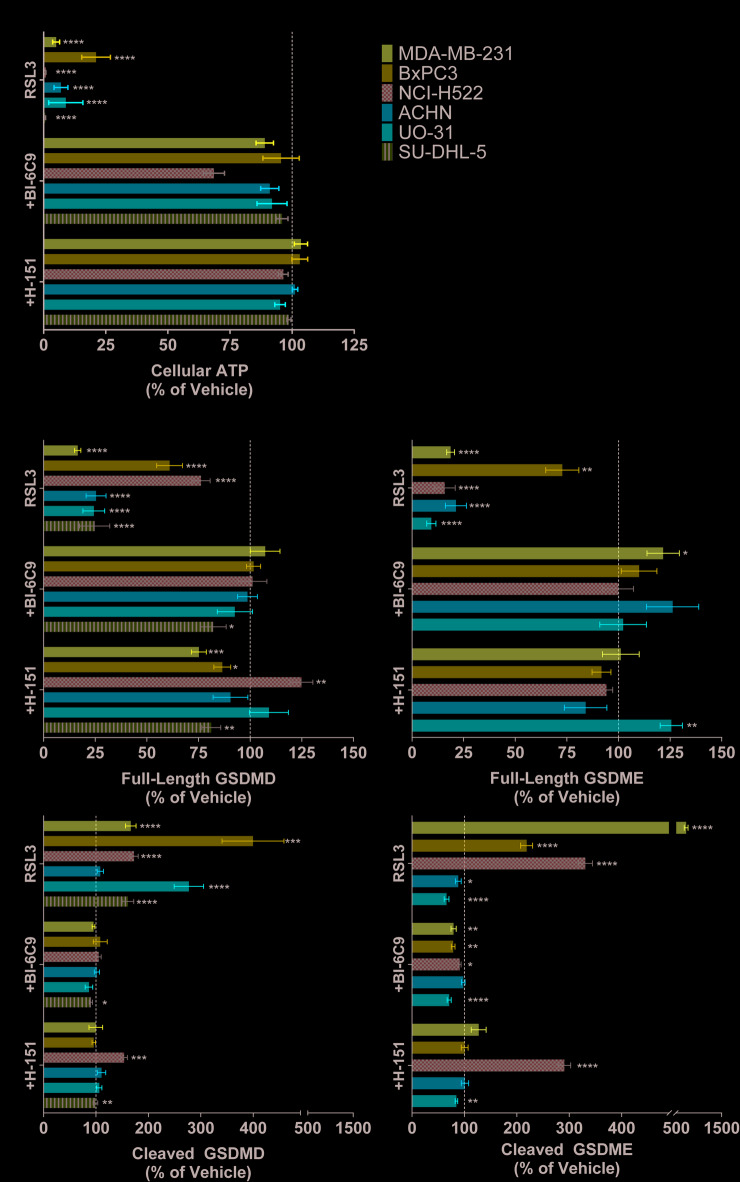
BID Activation and STING Signaling Are Critical in RSL3-induced Pyroptosis. (A) ATP levels relative to vehicle control following 1 day of RSL3 treatment with or without STING inhibitor (H-151) or BID inhibitor (BI-6C9) co-treatment. All *N* ≥ 3. Error bars: SEM. (B) Full-length and cleaved GSDMD and GSDME levels relative to vehicle control following 1 day of RSL3 treatment with or without H-151 or BI-6C9 co-treatment. All *N* ≥ 3. Error bars: SEM. Probability values: * *p* < 0.05; ** *p* < 0.01; *** *p* < 0.001; **** *p* < 0.0001.

We next investigated the involvement of BID and STING signaling in RSL3-induced cytotoxicity due to their reported roles in both ferroptosis^42,43^ and pyroptosis^44,45^. In all 6 cell lines tested, inhibitors of BID (BI-6C9) or STING (H-151) prevented the deleterious effects of RSL3 on ATP levels (Fig. 3A) and the appearance of necrotic cells (Supplemental Fig. S6). Inhibiting either pathway also reduced GSDMD/E cleavage to vehicle levels in all cell lines, except for NCI-H522 cells, in which STING inhibition only slightly reduced GSDMD and GSDME cleavage despite restoration of full-length protein levels (Fig. 3B). We note that 3 of the 6 cell lines in our study express very little STING (NCI-H522, ACHN, and SU-DHL-5), and 2 cell lines express no measurable cGAS protein (ACHN, UO-31) on Western blot (Supplemental Fig. S7; see also Supplemental Materials for complete WB pictures). Although BID inhibition effectively prevented cell death from RSL3 we did not detect truncated BID in RSL3-treated BxPC3 cells by Western blot (data not shown); however, multiple studies have reported that BID is involved in ferroptosis and full-length BID can localize to the outer mitochondrial membrane in a process that is inhibited by BI-6C9^42,46^.

Both inhibitors prevented RSL3-induced loss of GPx4 (Supplemental Fig. S8) to > 80% of vehicle levels in UO-31 and SU-DHL-5 cells (*p* < 0.05), but had little effect in MDA-MB-231 cells and no effect in BxPC3 and ACHN cells. In NCI-H522, the only cell line where GPx4 protein levels did not decrease following RSL3 treatment, these inhibitors nearly doubled (BID inhibitor) or tripled (STING inhibitor) GPx4 protein levels relative to vehicle (*p* < 0.05, Supplemental Fig. S8). Both inhibitors partially or completely prevented TfR modulation caused by RSL3 treatment in 4/6 cell lines (Supplemental Fig. S8). Both BID and STING inhibition blocked RSL3-induced caspase-3 activation in MDA-MB-231 and SU-DHL-5 cells and partially prevented loss of active caspase-3 in UO-31 cells (Supplemental Fig. S8). Only the STING inhibitor partially prevented loss of active caspase-3 in BxPC3 and induced caspase-3 activation in NCI-H522 cells (Supplemental Fig. S8).

### RSL3 induces secretion of pyroptosis-associated cytokines

**Fig. 4:**
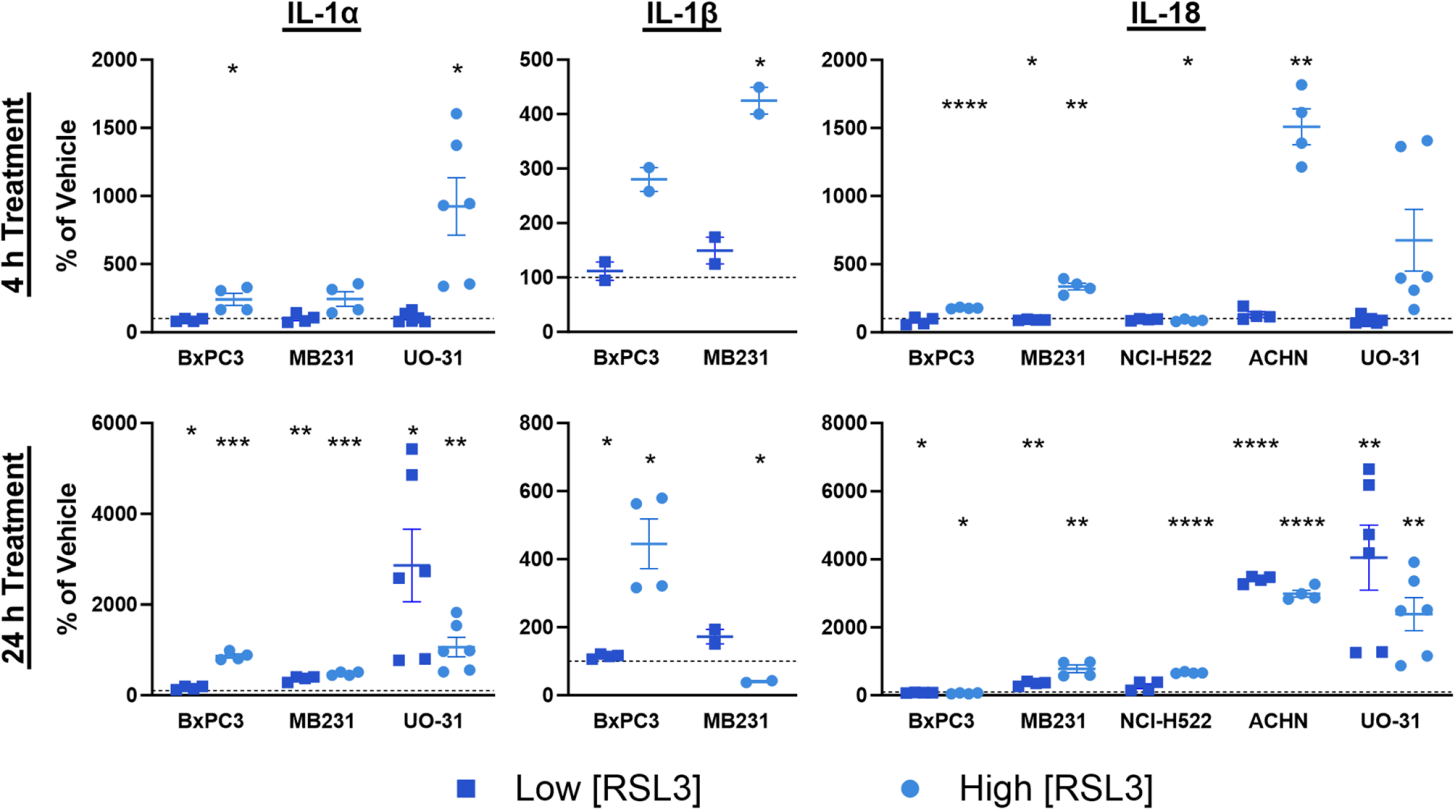
RSL3 Induces Secretion of Pyroptosis-Associated Cytokines. Cells treated with 2 concentrations of RSL3 (see Supplemental Table S1) for 4 h and 24 h secrete at least 1 pyroptosis-associated cytokine in all but the SU-DHL-5 cell line. *N* ≥ 3 except for IL-1β plots where *N* = 2 for 4 h treatments (MDA-MB-231 and BxPC3) and 24 h treatments (MDA-MB-231) due to IL-1β being below detection limits in vehicle samples. Error bars: SEM. Probability values: * *p* < 0.05; ** *p* < 0.01; *** *p* < 0.001; **** *p* < 0.0001.

We used a commercial Luminex assay to measure pyroptosis-associated cytokines (IL-1α, IL-1β, IL-18)^17,47^ in cell culture supernatant after 4 h and 24 h of RSL3 treatment (a ‘high’ RSL3 concentration used in all experiments and a ‘low’ concentration only used in cytokine experiments; see Supplemental Table S1). Increased IL-18 was detected in the supernatants from RSL3-treated cells of all cell lines except SU-DHL-5, IL-1α was detected in 3 cell lines (BxPC3, MDA-MB-231, UO-31) and IL-1β in 2 cell lines (BxPC3, MDA-MB-231) (Fig. 4). RSL3-induced cytokine secretion was greatest for ACHN (IL-18) and UO-31 (IL-1α, IL-18).

## Discussion

Vulnerability to ferroptosis has been identified in multiple therapy-resistant cancer cells^12,48^, making this pathway an attractive target for drug development. However, better tools are required to more precisely understand overlapping cell death pathways. In this study, we apply novel multiplex immunoassays to interrogate biomarkers of ferroptosis and pyroptosis and provide evidence that a commonly used ferroptosis inducer, the GPx4 inhibitor RSL3, also induces pyroptosis in cancer cells. Our data show that RSL3 induced the appearance of necrotic cells (Fig. 1A), cleavage of both GSDMD/E (Fig. 2B), and release of pyroptosis-associated cytokines (Fig. 4). The results are summarized in Table 1. Reversal of both ATP depletion and GSDMD/E cleavage following treatment with BID and STING inhibitors (Fig. 3) suggests that mitochondria may link ferroptosis and pyroptosis following RSL3-induced cell death, but further work is needed to distinguish the relative contributions and time courses followed by the 2 independent cell death pathways.

**Table 1:** Summary of results for cell culture multiplex assays following RSL3 treatment with/without cell death pathway inhibitors.

Cell Line	RSL3-Induced			RSL3-Induced Cell Death Prevented^1^ By				
	Cell Death	Gasdermin Cleavage	IL-1/IL-18 Secretion	Fer-1	zVADfmk	Nec-1	BID i	STING i
MDA-MB-231	95%	GSDMD GSDME	Yes	Yes	No	Partial	Yes	Yes
BxPC3	79%	GSDMD GSDME	Yes	Yes	Partial	Partial	Yes	Yes
NCI-H522	99%	GSDMD GSDME	Yes	Yes	No	Partial	Partial	Yes
ACHN	93%	GSDMD^2^	Yes	Yes	No	Partial	Yes	Yes
UO-31	91%	GSDMD	Yes	Yes	No	No	Yes	Yes
SU-DHL-5	99%	GSDMD	ND^3^	Yes	No	Yes	Yes	Yes

1. Yes: cell death < 25% with inhibitor; No: cell death > 75% with inhibitor; Partial: 25% < cell death with inhibitor < 75%.

2. Cleavage only detected when RSL3 is combined with zVADfmk.

3. Not detected.

A swollen, balloon-like morphology has been historically associated with unregulated necrosis^49^, which in ferroptosis is assumed to be caused by nanopores formed when phospholipid peroxidation damages the plasma membrane^50^. Pores formed during ferroptosis^25,51^ and pyroptosis^20,52^ are similar in size. Distinguishing between forms of cell death therefore requires evidence of involvement of pathway-specific activated protein executioner(s), e.g., cleaved gasdermins (for pyroptosis), phosphorylated or oligomerized Mixed Lineage Kinase Domain Like (MLKL, for necroptosis), and blebbing-localized cleaved caspase-3 (for apoptosis). No protein executioner biomarker has been described for ferroptosis, but recent research suggests protein lipid modifications are attractive targets for biomarker development^53^.

Using the common approach of combining a cytotoxic compound (RSL3) with inhibitors of lipid oxidation (Fer-1), caspases (zVADfmk), or necroptosis (Nec-1) to identify the cell death pathway, we observe a pattern consistent with ferroptosis: ATP depletion was completely prevented by Fer-1, not significantly blocked by zVADfmk, and the effect of Nec-1 varied by cell line (Fig. 2A). However, we have not detected evidence of necroptosis by Western blot in select cell lines where Nec-1 either partially (NCI-H522) or markedly (ACHN, SU-DHL5) blocked cell death (Supplemental Fig. S9; see also Supplemental Materials for complete WB pictures). A recent report concluded that Nec-1 inhibits ferroptosis by upregulating antioxidant genes, rather than by inhibiting RIPK1 or indoleamine 2,3-dioxygenase 1/2 (IDO1/2)^13^, suggesting that Nec-1 is not a useful tool for distinguishing necroptosis from other forms of cell death.

Our biomarker data suggest that the role of caspases in RSL3-induced ferroptosis and in pyroptosis may be more complex than previously reported. Unexpectedly (but not unprecedented^54^), RSL3 induces caspase-3 activation in 2 cell lines (Supplemental Fig. 1), which along with GSDME cleavage, is inhibited by zVADfmk. GSDME cleavage is also inhibited in cell lines without RSL3-induced caspase-3 activation. This suggests that GSDME may be cleaved by other proteases sensitive to zVADfmk, or that caspase-3 activity is altered in the context of excessive lipid peroxidation through protein modifications such as carbonylation, glutathionylation, etc. Paradoxically, caspase inhibition dramatically increases GSDMD cleavage in 3 cell lines (Fig. 2B); the mechanisms remain elusive, but a possible explanation is that caspase inhibition relaxes a negative feedback signal on an unidentified protease. Recent studies have identified non-caspase proteases capable of cleaving GSDMD or GSDME^55–57^. Interestingly, caspase inhibition by zVADfmk has been reported to increase intracellular ROS in glutamate-induced neurotoxicity, a form of oxytosis/ferroptosis^58^. Finally, recent studies have reported either an association between ferroptosis and pyroptotic gene expression^59^ or promotion of GSDMD-N oligomerization by cysteine oxidation^60^ or lipid peroxidation^33,61^, providing a potential link between ROS and gasdermin activation.

The N-terminal domains of cleaved gasdermins oligomerize to form pores in the cell membrane leading to the release of inflammatory molecules and pyroptotic cell death^21^. Here we report the release of at least 1 pyroptosis-associated interleukin (IL-1α, IL-1β, IL-18) in 5/6 cell lines following treatment with the ferroptosis inducer RSL3 (Fig. 4; Table 1). SU-DHL-5, the only cell line where we do not detect interleukin release, also does not express detectable levels of GSDME and experiences limited GSDMD cleavage compared to other cell lines following RSL3 treatment. High levels of secreted interleukins correlate with increased GSDMD and/or GSDME cleavage in 3/5 cell lines (UO-31, BXPC3 and MDA-MB-231), whereas increased GSDMD/E cleavage does not correlate with substantial release of interleukins following RSL3 treatment in NCI-H522 cells. Overall, our results are consistent with pyroptotic cell death following RSL3 treatment, although the exact mechanism of action remains to be determined.

Our findings confirm prior studies reporting involvement of STING and the apoptosis-associated protein BID, in ferroptosis^46,62^ and pyroptosis^28,30^. ATP depletion by RSL3 is almost completely prevented by inhibiting either of these pathways (Fig. 3A), independent of the variable levels of STING protein in the cell lines. Both inhibitors restore full-length GSDMD/E levels to approximate vehicle controls compared to RSL3 treatment. The inhibitors also prevent cleavage of GSDMD in 4/6 cell lines, and of GSDME in 3/5 cell lines (Fig. 3B). We believe that STING may connect pyroptosis and ferroptosis through lysosomes and/or the endoplasmic reticulum^28,62,63^ culminating in the activation or release of proteases such as calpains^58,64,65^ or cathepsins^57,65,66^.

Overall, although our results strongly indicate that pyroptosis is induced in cancer cells following RSL3 treatment, the relative contributions of ferroptosis and pyroptosis to RSL3-induced cell death is currently unknown. Similar claims were made in a recent study which demonstrates GSDMD cleavage in RSL3-treated neuron-like rat cells and brain tissue^67^. Our study emphasizes the critical need to develop reliable biomarkers that better distinguish between different cell death pathways. Increased TfR expression is reported to be a specific biomarker for ferroptosis^8^, but our data do not support this finding. Based on our observations, we recommend using a combination of morphology and measurement of pathway-specific protein biomarkers with validated antibodies or reagents to identify the mechanism of action of novel cytotoxic agents. When small molecule inhibitors are used, it is critical to consider their concentrations and possible off-target effects; such observations need to be corroborated by additional evidence to facilitate translational drug development for patients with cancer and other diseases.

## Electronic supplementary material

Below is the link to the electronic supplementary material.

Supplementary Material 1

## Data Availability

The original data presented in the study are included in the article/supplementary material. For further inquiries contact the corresponding authors.
