# Supplementary material for: Potent ferroptosis agent RSL3 induces cleavage of Pyroptosis-Specific gasdermins in Cancer cells
Source: Sci Rep. 2025 Jul 12;15:25249. doi: 10.1038/s41598-025-11368-1 (PMC12255707; doi:10.1038/s41598-025-11368-1)
Supplement: Supplementary file 1 — Supplementary Material 1 [file 41598_2025_11368_MOESM1_ESM.docx]

# Supplemental Methods

*Western Blotting*

Whole cell lysates were diluted with ddH_2_O and denatured with Laemmli sample buffer (Bio-Rad) under reducing conditions (boiled for 5-10 min in presence of 2.5% β-mercaptoethanol), then separated by electrophoresis on precast 4-20% Mini-PROTEAN TGX SDS-PAGE gels (20 µg/lane; Bio-Rad). Proteins were transferred to nitrocellulose membranes with a Trans-Blot Turbo apparatus (Bio-Rad) and blocked for 1 hour with LI-COR TBS Intercept Blocking Buffer. Blots were incubated with antibodies diluted in blocking buffer containing 0.1% Tween 20 (Roche) overnight at 4°C or at room temperature for 2 hours. Detection was performed with LI-COR fluorescent secondary antibodies, and blots were imaged with a LI-COR Odyssey M imaging system~~.~~ and images were processed in LI-COR’s Image Studio 6.0 for visibility and clarity~~.~~ Primary antibodies were obtained from Cell Signaling Technologies (GSDMD clone E8G3F [Suppl. Fig. S3], STING clone D2P2F, cGAS clone E5V3W, MLKL clone D2I6N, and COX IV clone 4D11-B3-E8), Abcam (GSDME clone EPR19859, phospho-MLKL clone EPR9514, and GAPDH clone EPR6256), and ProteinTech (GSDMD clone 2E6C7 [Suppl. Fig. S5]). LI-COR NewBlot Nitrocellulose Stripping Buffer was used to strip the phospho-MLKL antibody from blots before re-probing for total MLKL.

*Luminex Assay Antibodies and Recombinant Protein Calibrators*

We expressed recombinant protein calibrators in *E. coli* (full-length GSDMD[2-484], cleaved GSDMD[2-275], full-length GSDME[1-496]), HEK293E cells (GPx4[26-197]), or Tni-FNL insect cells (cleaved GSDME[1-270]). All proteins were N-terminally tagged with His6-MBP-tev and purified by IMAC. For simplicity, selenocysteine-73 in GPx4 was substituted with Cys, and the cleaved GSDMD yield was increased with an I104N substitution (1). Purity was assessed by SDS-PAGE and protein concentrations were measured using the Bradford method (Bio-Rad). The protein calibrator for active caspase-3 was purchased from R&D Systems. No protein calibrator was used for full-length transferrin receptor (TfR) due to lack of commercial products or published methods to recombinantly produce full-length TfR protein.

We developed highly specific monoclonal antibodies targeting the neoepitopes of cleaved GSDMD and GSDME (pore-forming N-terminal fragments) for sandwich immunoassay (2); all other antibodies used in our Luminex assay are commercially available monoclonal antibodies from Cell Signaling Technologies, Abcam, Santa Cruz Biotech, Proteintech, Invitrogen, or R&D Systems. Detection antibodies were conjugated to biotin using Sulfo-NHS-LC-Biotin (Thermo Fisher Scientific) and excess biotin was removed with Zeba Spin Desalting columns (Thermo Fisher Scientific) according to manufacturer’s instructions. Capture antibodies were conjugated to MagPlex microspheres (Luminex) with standard EDC and sulfo-NHS (Thermo Fisher Scientific) chemistry.

*Luminex Multiplex Immunoassays*

Our ferroptosis/pyroptosis immunoassay was developed on the Luminex xMAP technology platform using MagPlex® polystyrene-coated magnetic beads (Luminex) and were performed in flat-bottomed black 96-well plates (Bio-Rad) protected from light with black microplate lids (VWR) during incubations. Sample lysates with a total protein concentration > 1 mg/mL were diluted to 1 mg/mL in assay buffer; samples with total protein concentration < 1 mg/mL were undiluted. An equivalent volume of denaturation buffer was added to each sample, which were subsequently incubated on ice for 1 hour, then diluted further by addition of an equal volume of assay buffer. Finally, the denatured and diluted samples were frozen on dry ice, then thawed on wet ice immediately before loading assay plates. Components were added to the assay plates as follows: 10 μL/well blocking buffer, 30 μL/well of sample/standard/control/blank, and 10 μL/well bead cocktail. Plates were incubated for 16–18 hours at 4 ± 3°C with shaking (850 rpm). Plates were then washed 3 times using a BioTek 405 TS plate washer with 300 μL/well of wash buffer. After washing, 50 μL/well of antibody-biotin conjugate in detection buffer was added and the plates were incubated with shaking for 1 hour at room temperature. Plates were washed again as described above. Premium grade R-phycoerythrin-labeled streptavidin (Invitrogen, 50 µL/well) was added, and the plates were incubated with shaking for 30 minutes at room temperature. Plates were washed again, then 100 μL/well of wash buffer was added and plates were incubated with shaking for 1–2 minutes to resuspend beads. Plates were read on Luminex 200 instruments with xPONENT software.

*Analytical Validation*

Assay analytical validation was performed to assess reproducibility, precision, and dilutional linearity/recovery (2).

Cleaved GSDMD peptide competition assay (PCA) was performed by assaying samples with and without the same peptide immunogen used for antibody generation (unconjugated; Biosynth). The peptide was added to the assay buffer to approximately 200-fold greater concentration than the cleavage-specific capture antibody.

# Supplemental Tables

**Supplemental Table S1:** Cell density information and RSL3 concentration

| **Cell Line** | **Cell Type** | **Low [RSL3] (nM)** | **High [RSL3] (nM)*** | **T-175 Seeding Density**  **(cells x10^6^)** | **Plate Seeding Density**  **(cells/well)** |
| --- | --- | --- | --- | --- | --- |
| MDA-MB-231 | Triple negative breast cancer | 100 | 500 | 1.2 | 24,000 [24 well plate]  8,000 [96 well plate] |
| BxPC3 | Pancreatic cancer | 200 | 500 | 1.4 | 24,000 [24 well plate]  8,000 [96 well plate] |
| NCI-H522 | Non-small cell lung cancer | 25 | 100 | 2 | 40,000 [24 well plate]  6,000 [96 well plate] |
| ACHN | Renal cancer | 100 | 500 | 3 | 200,000 [12 well plate]  20,000 [96 well plate] |
| UO-31 | Renal cancer | 25 | 100 | 0.8 | 60,000 [12 well plate]  6,000 [96 well plate] |
| SU-DHL-5 | Diffuse large B-cell lymphoma | 25 | 100 | 2.5x10^5^ cells/mL | 250,000 [12 well plate]  25,000 [96 well plate] |

* the concentration of RSL3 used in biomarker and CellTiter Glo assays.

**Supplemental Table S2:** Compound source and concentrations used in biomarker experiments

| **Reagent** | **Purpose** | **Source** | **Identifier** | **Test Concentration** |
| --- | --- | --- | --- | --- |
| RSL3 | Ferroptosis induction | NCI Developmental Therapeutics Program | NSC 833292 | 25 – 1000 nM |
| Ferrostatin-1 | Ferroptosis inhibition | Selleckchem | S7243 | 2 µM |
| zVADfmk | Caspase inhibition | Selleckchem | S7023 | 50 µM |
| BI-6C9 | BID inhibition | MedChem Express | HY-103661 | 10 µM |
| H-151 | STING inhibition | MedChem Express | HY-112693 | 2 µM |
| Necrostatin-1 | Necroptosis inhibition | EMD Millipore | 480065 | 25 µM |
| Emricasan | Inhibit caspases | Selleckchem | S7775 | 10 µM |
| TNFα | Activate NF-kB pathway | Abcam | ab259410 | 20 ng/mL |
| Tolinapant | Inhibit IAPs | NCI Developmental Therapeutics Program | NSC 804861 | 1 µM |

# Supplemental figures and Figure legends:

**Supplemental Figure 1:** GPx4, TfR, and active caspase-3 levels relative to vehicle control following 1 day of RSL3 treatment with or without ferroptosis inhibitor (Fer-1), caspase inhibitor (zVADfmk) or necroptosis inhibitor (Nec-1). Active caspase-3 levels were below the lower limit of quantification (LLQ) in all the measured ACHN samples. All N ≥ 3. Error bars: SEM.


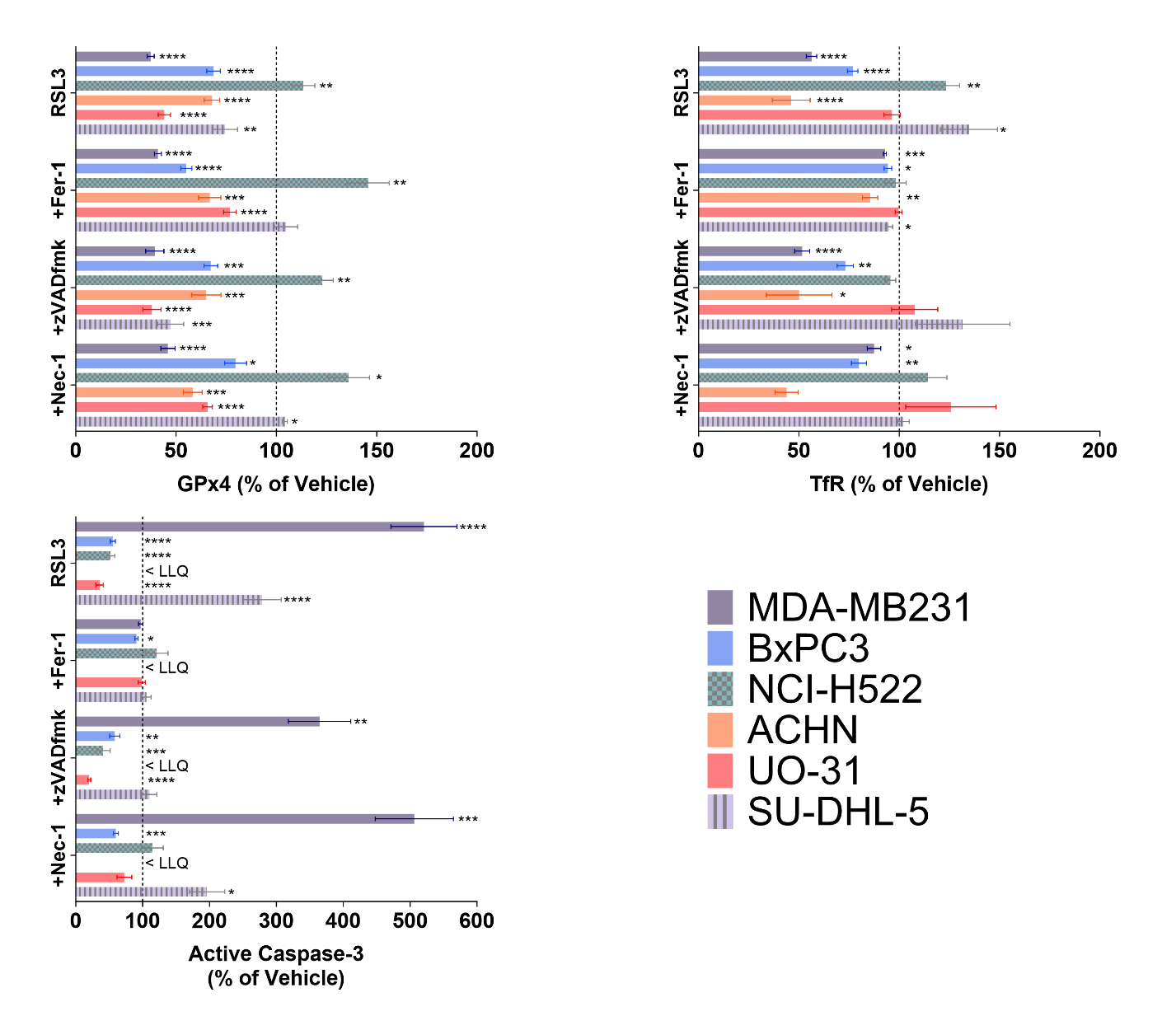


**Supplemental Figure S2:** RSL3-induced necrotic morphologies are not inhibited by zVADfmk or Nec-1. Scale bars for SU-DHL5: 100 µm; all other scale bars: 200 µm.

**
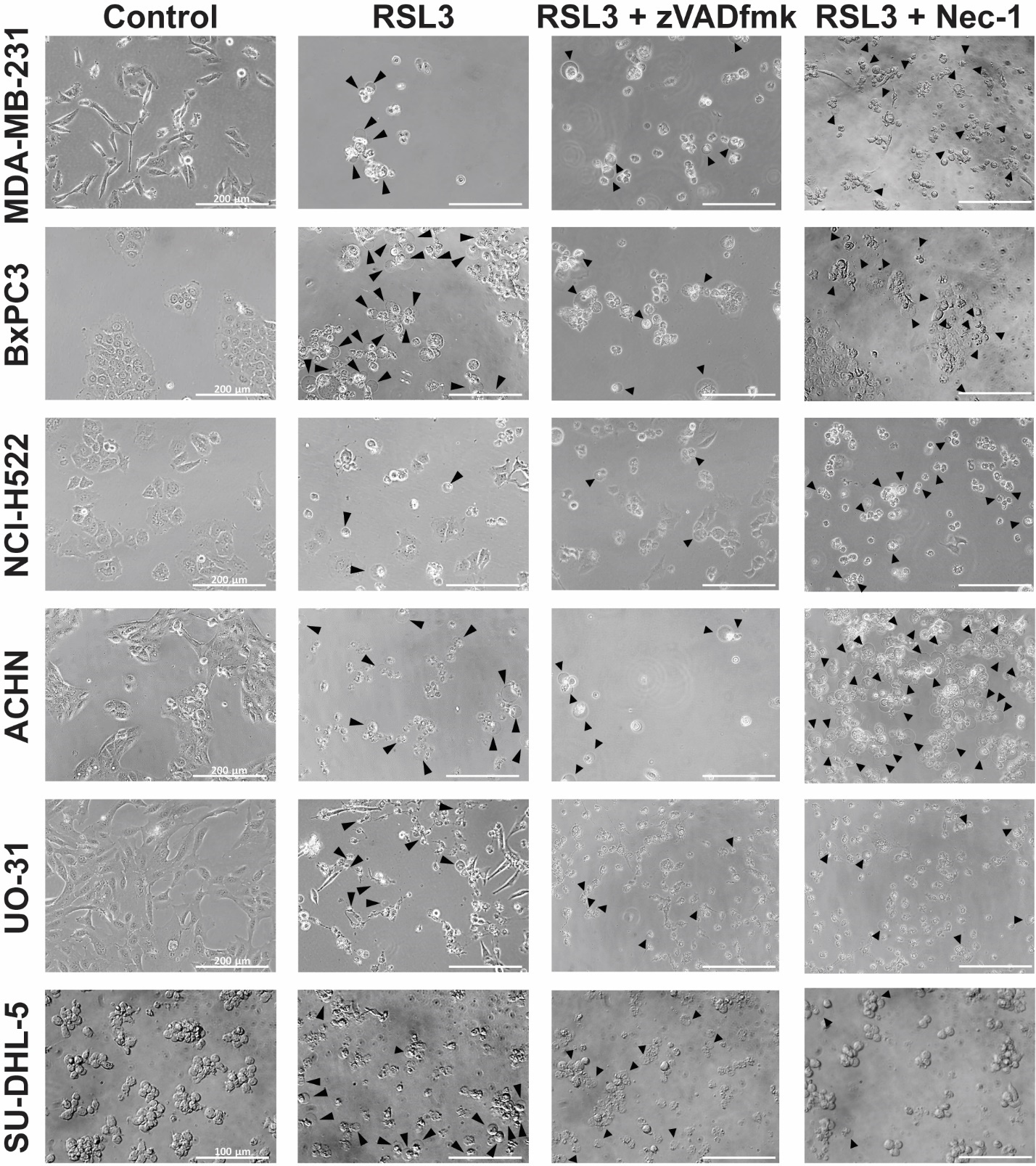
**

**Supplemental Figure S3:** Comparison of Western blot and Luminex assay quantification of baseline expression of full-length GSDME (A) and full-length GSDMD (B). The same sample lysates were used for both assays to allow direct comparison. Luminex data and Western blot band intensities were normalized against the highest-expressing cell line (UO-31 for GSDME, SU-DHL5 for GSDMD). Cleaved GSDME (GSDME-N) is present at low levels in some cell lines. Western blot loading control: COX IV.


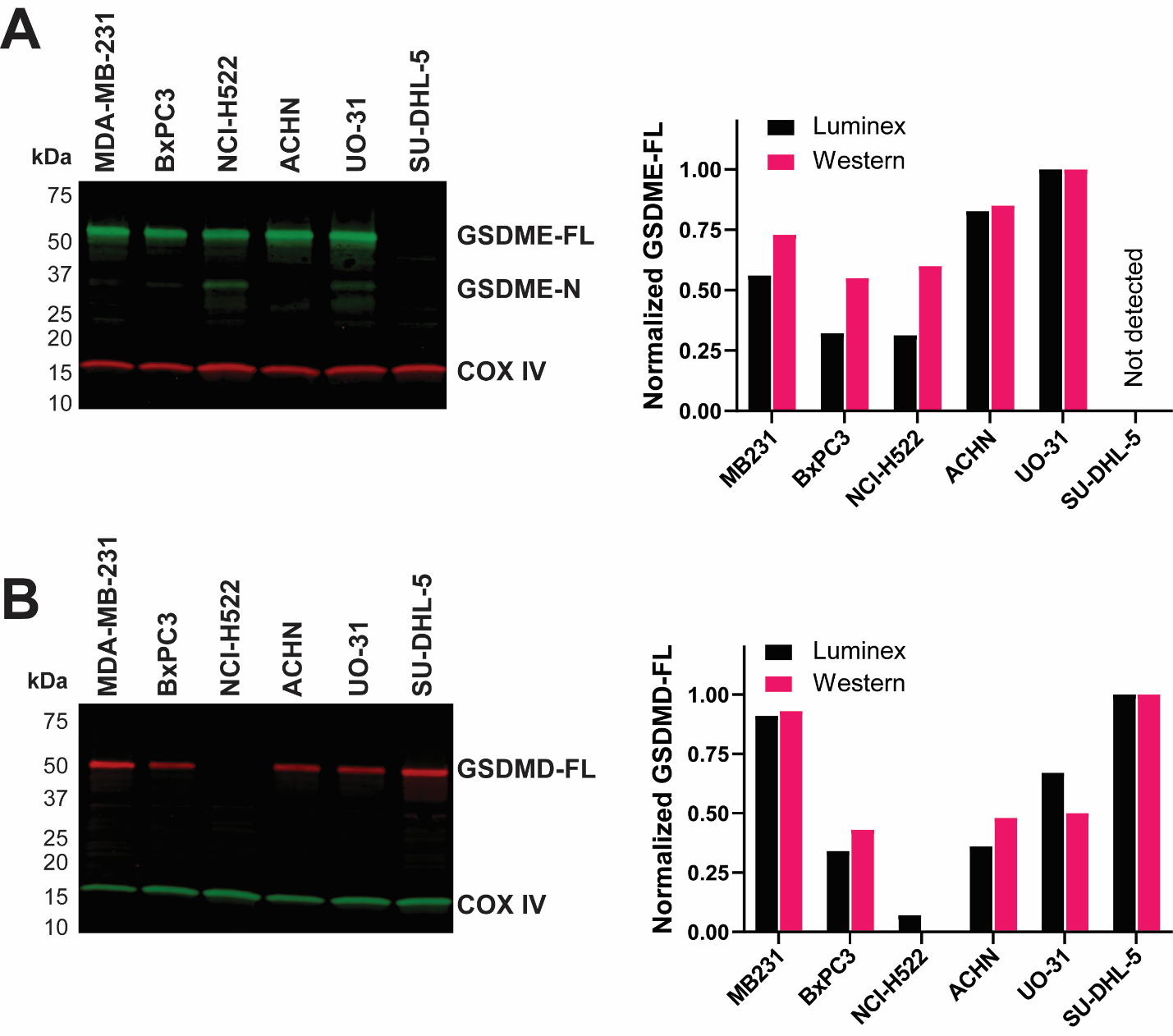


**Supplemental Figure S4:** Comparison of Western blot and Luminex quantification of full-length and cleaved GSDME in vehicle and RSL3-treated cell lysates. The same sample lysates were used for both assays to allow direct comparison. Luminex data and Western Blot band intensities were normalized to the vehicle average for each cell line. The COX IV loading control was measured on the same Western blots, after GSDME-N signal detection, following additional washes (no stripping).


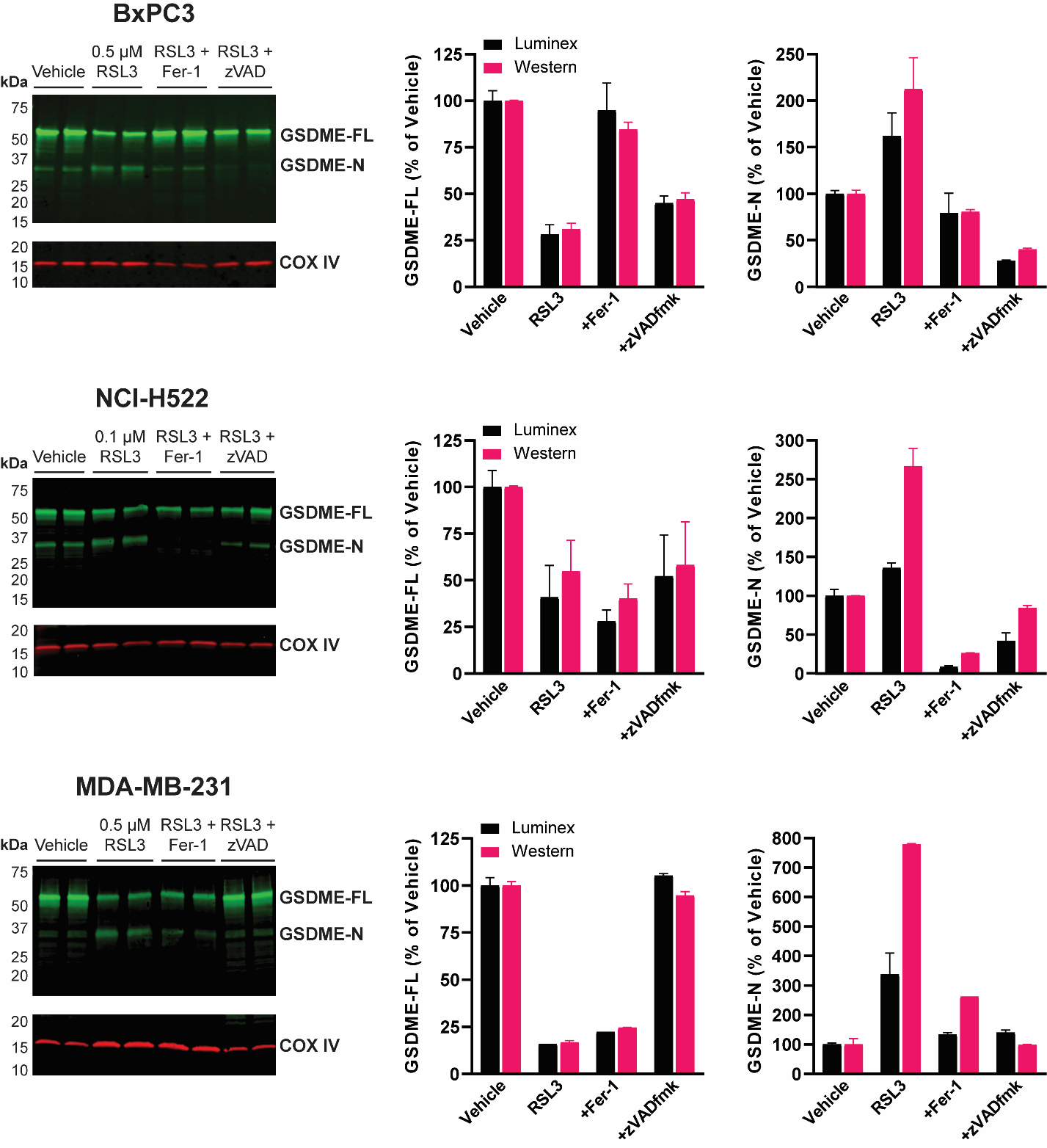


**Supplemental Figure S5:** An example of cleaved GSDMD detection via Western blot in the classical model of THP-1 macrophages primed with LPS and treated with 20 µM nigericin for 30 minutes (A) and Luminex assay results demonstrating the ability to detect loss of GSDMD-FL and gain of cleaved GSDMD in this model with comparison to BxPC3 cells treated with RSL3 and Nec-1 (B). Dashed and dotted red lines represent the limits of detection (LODs) for cleaved and full-length GSDMD, respectively, which were calculated by assaying 20 technical replicates of HAP1 GSDMD KO lysates and taking the mean values plus 3 standard deviations. Asterisks (*) denote non-specific bands in (A). Western blot loading control: GAPDH.


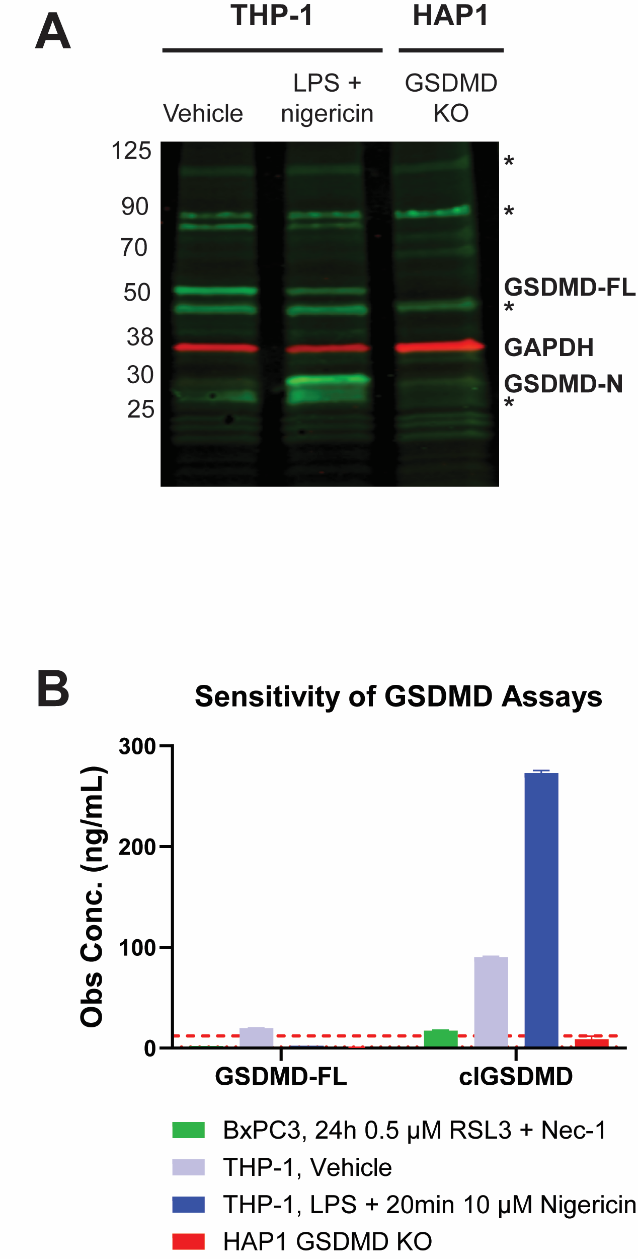


**Supplemental Figure S6:** RSL3-induced necrotic morphologies are inhibited by H-151 and BI-6C9. Scale bars for SU-DHL5: 100 µm; all other scale bars: 200 µm.


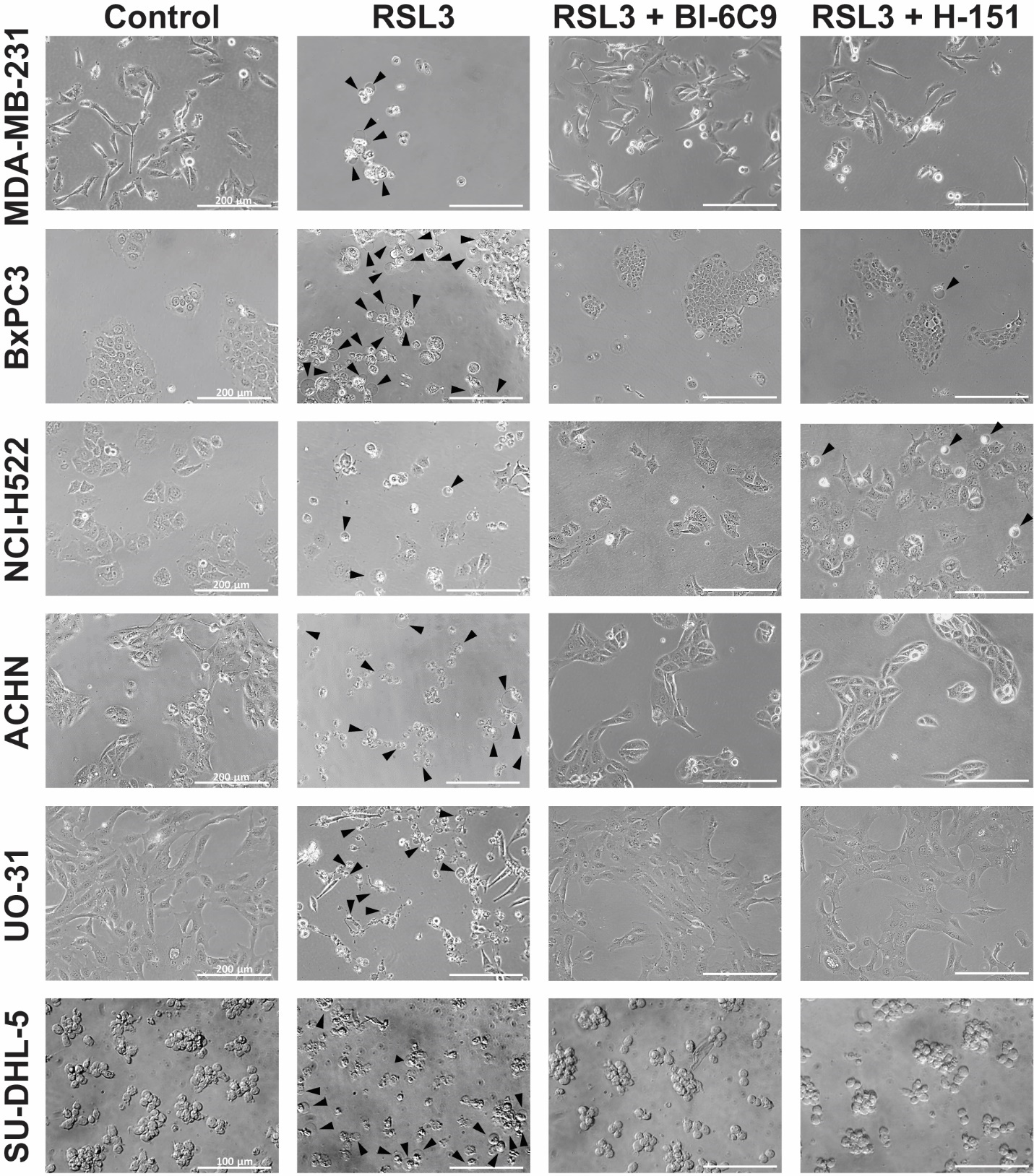


**Supplemental Figure S7:** Western blots showing STING (A) and cGAS (B) protein expression in cells treated with Vehicle or RSL3. A Vehicle-treated BxPC3 sample was used as a positive control for STING and cGAS expression. The same Western blots were probed for COX IV loading control (no stripping) and depicted separately due to COX IV higher signal intensities compared to STING or cGAS.


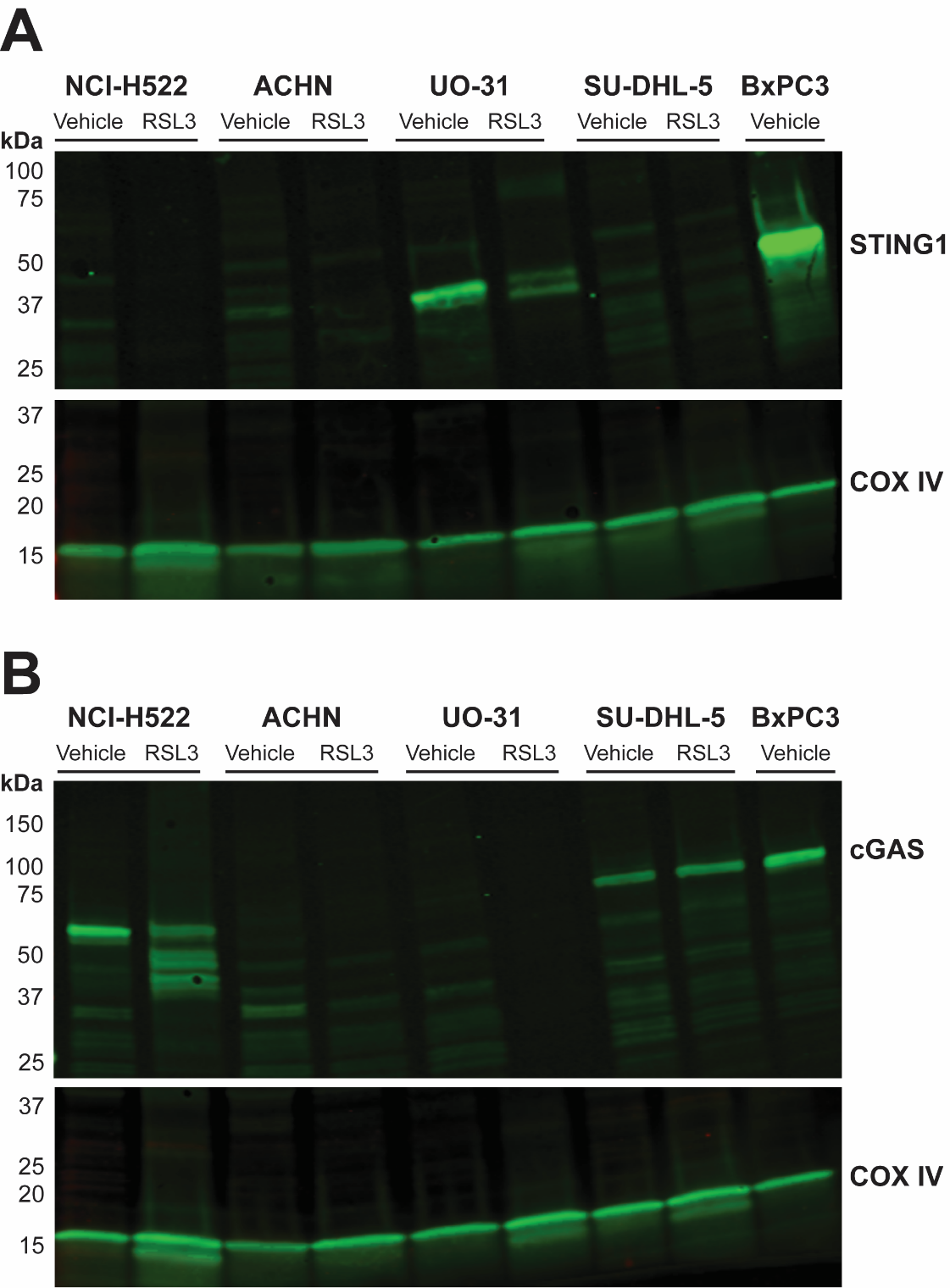


**Supplemental Figure S8:** GPx4, TfR, and active caspase-3 levels relative to vehicle control following 1 day of RSL3 treatment with or without BID inhibitor (BI-6C9) or STING inhibitor (H-151). Active caspase-3 levels were below the LLQ in all the measured ACHN samples. All N ≥ 3. Error bars: SEM.


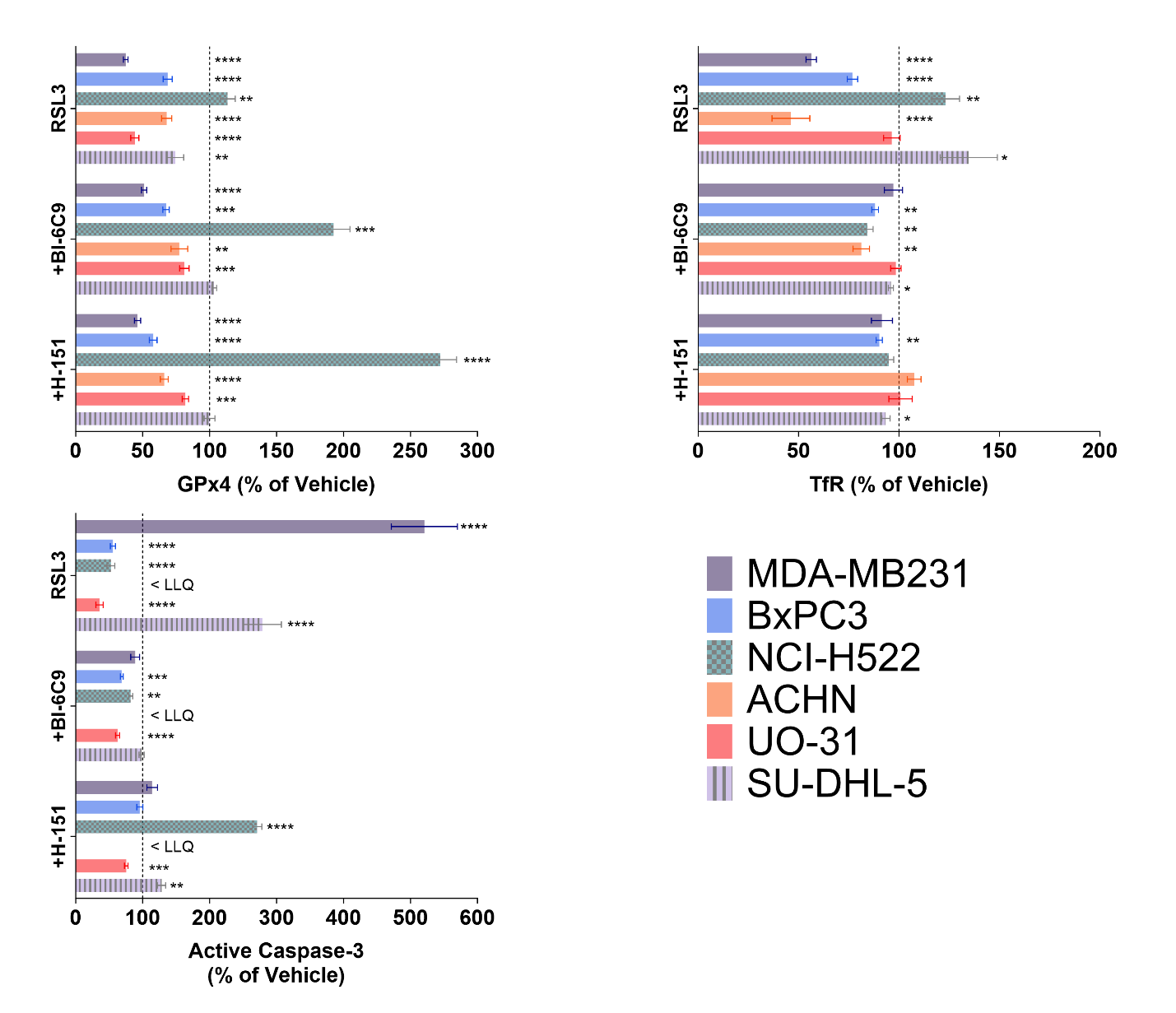


**Supplemental Figure S9:** (A) Western blot showing lack of MLKL phosphorylation in RSL3-treated NCI-H522, ACHN, and SU-DHL-5 cells with comparison to a well-known model of necroptosis (HT-29 colorectal cancer cells treated with TNFα, tolinapant, and emricasan). The green arrow identifies pMLKL in the positive control: treated HT-29 cell lysate. (B) The Western blots were stripped and re-probed with a total MLKL antibody. Asterisks (*) denote non-specific bands.


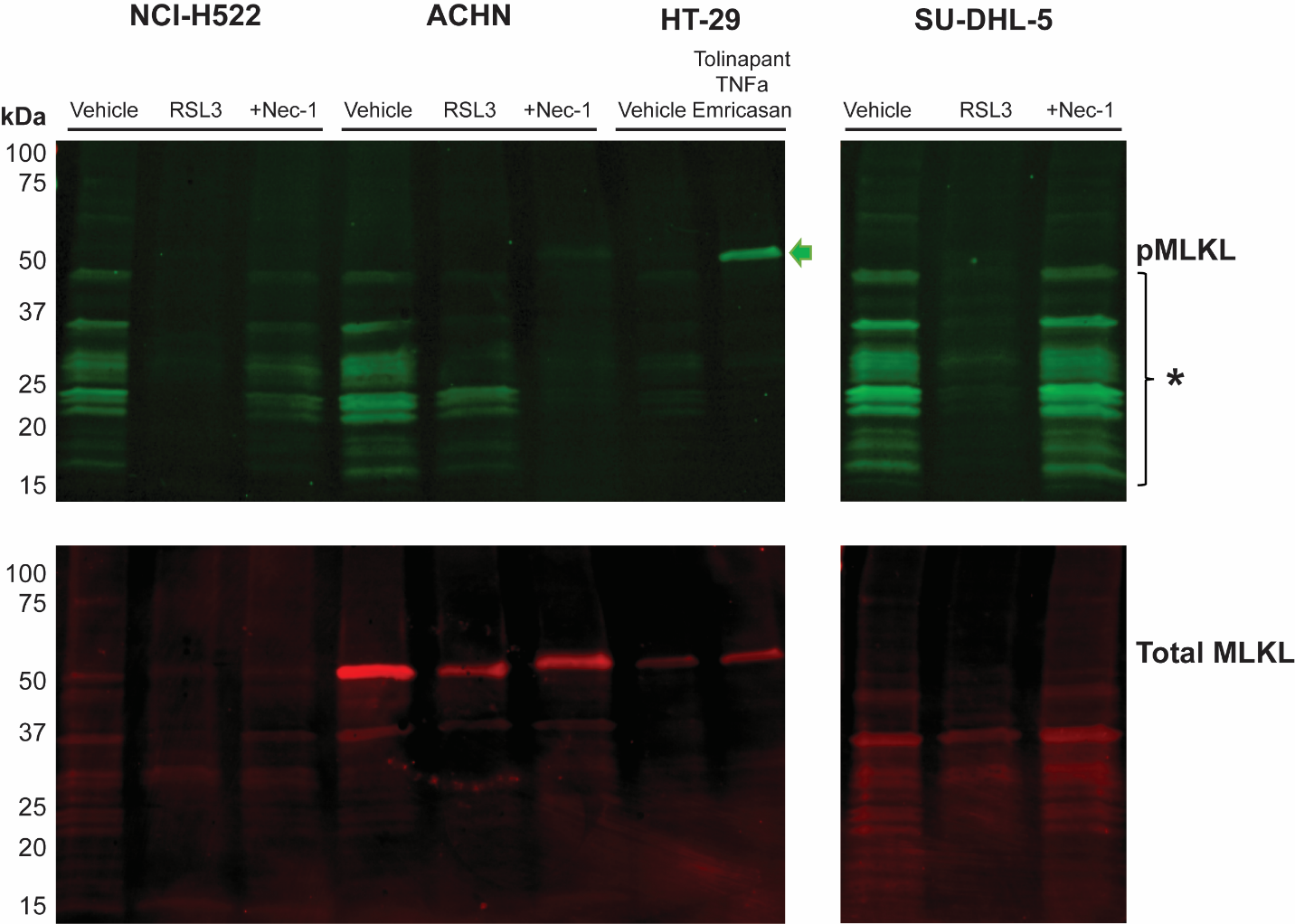


# Supplemental materials: unedited Western Blot Images

**Uncropped Western Blot Images for Supplemental Figure S3:**

A. 1. MDA-MB231; 2. BxPC3; 3. NCI-H522; 4. ACHN; 5. UO-31; 6. SU-DHL5; 7. Sample not included in this study. MW = molecular weight (BioRad Precision Plus Protein Dual Color Standards, #1610374).

MW 1 2 3 4 5 6 7 MW MW


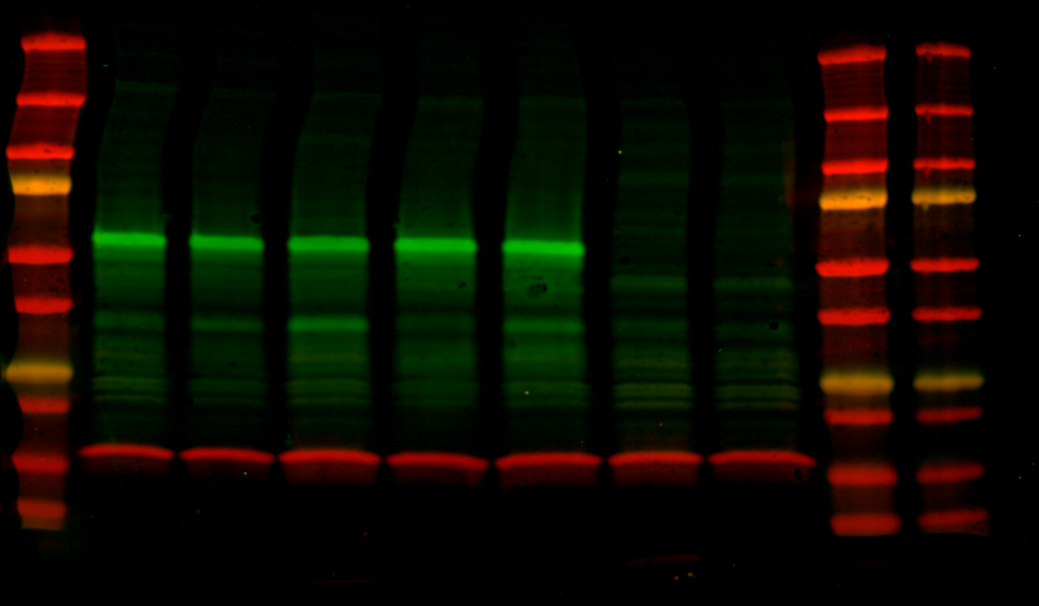


**GSDME-N**

**COX IV**

**GSDME-FL**

B. 1. MDA-MB231; 2. BxPC3; 3. NCI-H522; 4. ACHN; 5. UO-31; 6. SU-DHL5; 7-8. Samples not included in this study. MW = molecular weight (BioRad Precision Plus Protein Dual Color Standards, #1610374).

MW 1 2 3 4 5 6 7 8 MW


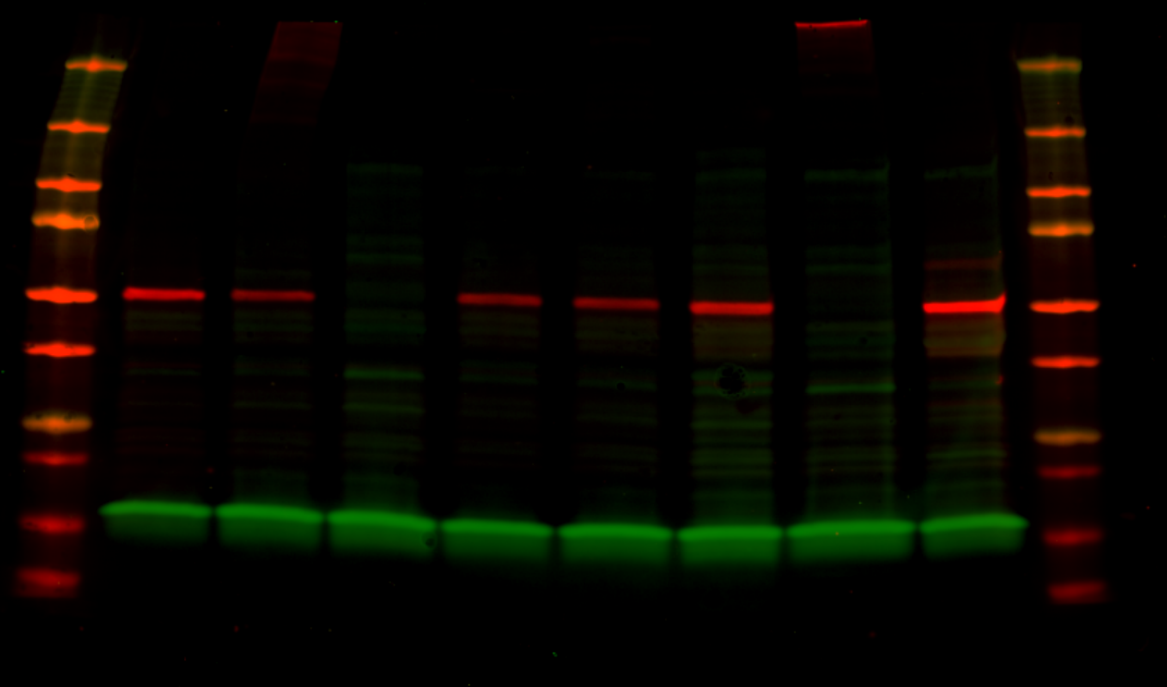


**COX IV**

**GSDMD-FL**

**Uncropped Western Blot Images for Supplemental Figure S4:**

**BxPC3:** 1-2. Vehicle; 3-4. 0.5µM RSL3; 5-6. RSL3 + Fer1; 7-8. RSL3 + zVADfmk; 9–12. Samples not included in this study. MW = molecular weight (BioRad Precision Plus Protein Dual Color Standards, Cat. No. 1610374).

Top: GSDME-N signal acquisition. Bottom: COX IV loading control measured on the same Western blots, after GSDME-N signal detection, following additional washes (no stripping).

MW 1 2 3 4 5 6 7 8 9 10 11 12 MW MW


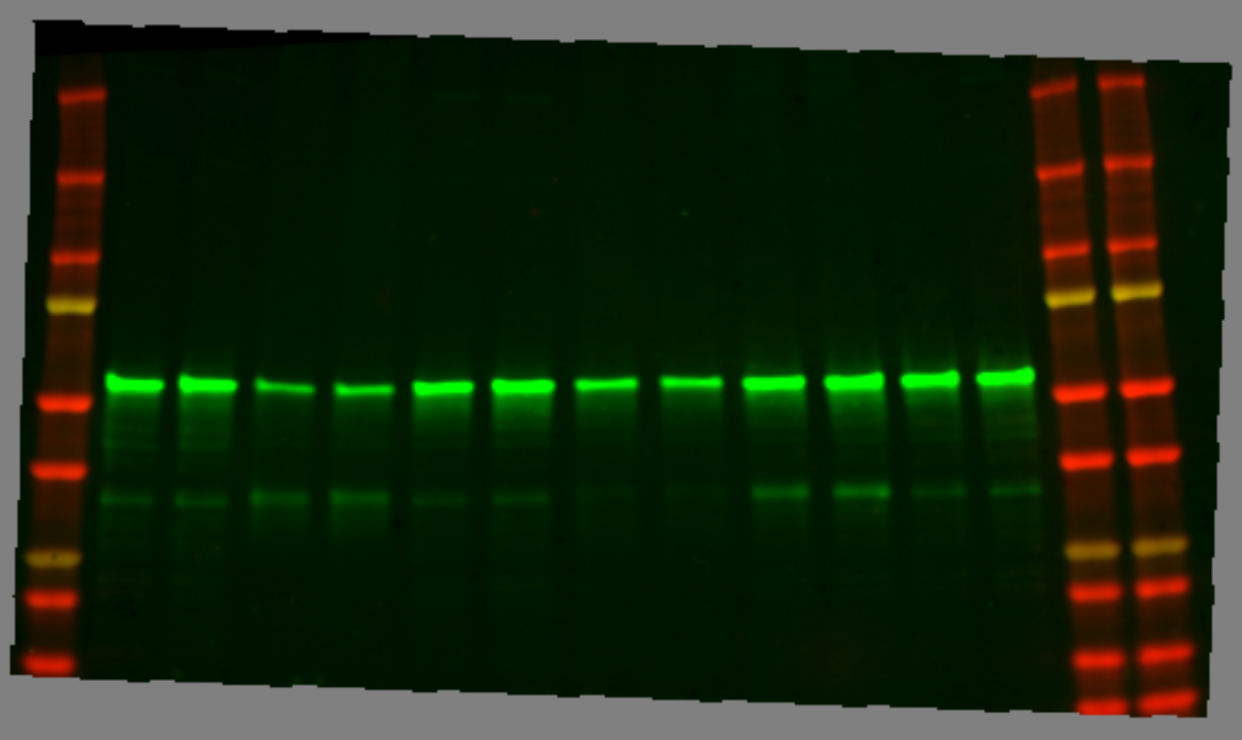


**GSDME-N**

**GSDME-FL**

MW 1 2 3 4 5 6 7 8 9 10 11 12 MW MW


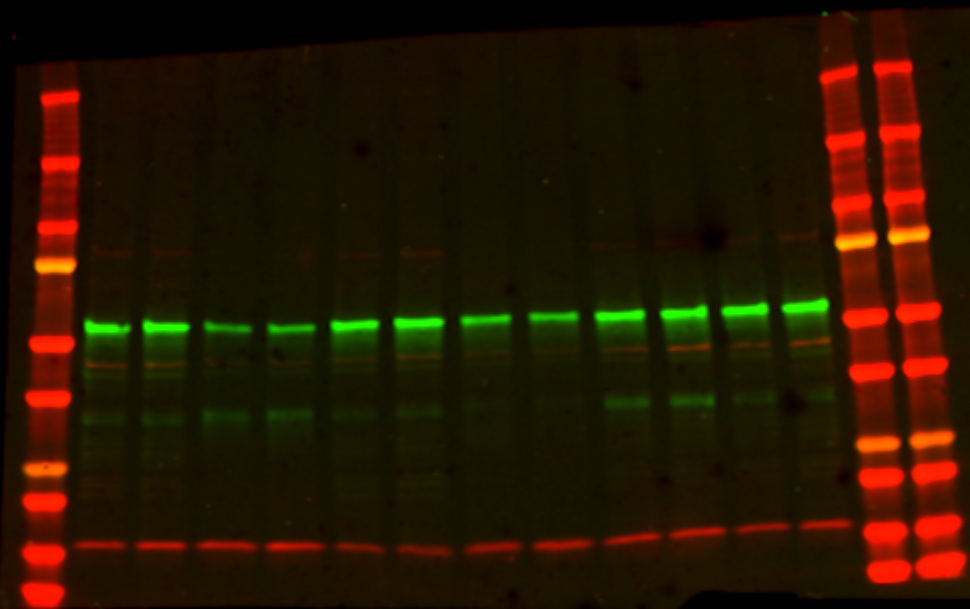


**GSDME-FL**

**GSDME-N**

**COX IV**

**NCI-H522:** 1-2. Vehicle; 3-4. 0.5µM RSL3; 5-6. RSL3 + Fer1; 7-8. RSL3 + zVADfmk; 9. Samples not included in this study. MW = molecular weight (BioRad Precision Plus Protein Dual Color Standards, Cat. No. 1610374).

Top: GSDME-N signal acquisition. Bottom: COX IV loading control measured on the same Western blots, after GSDME-N signal detection, following additional washes (no stripping).

MW 1 2 3 4 5 6 7 8 9

**
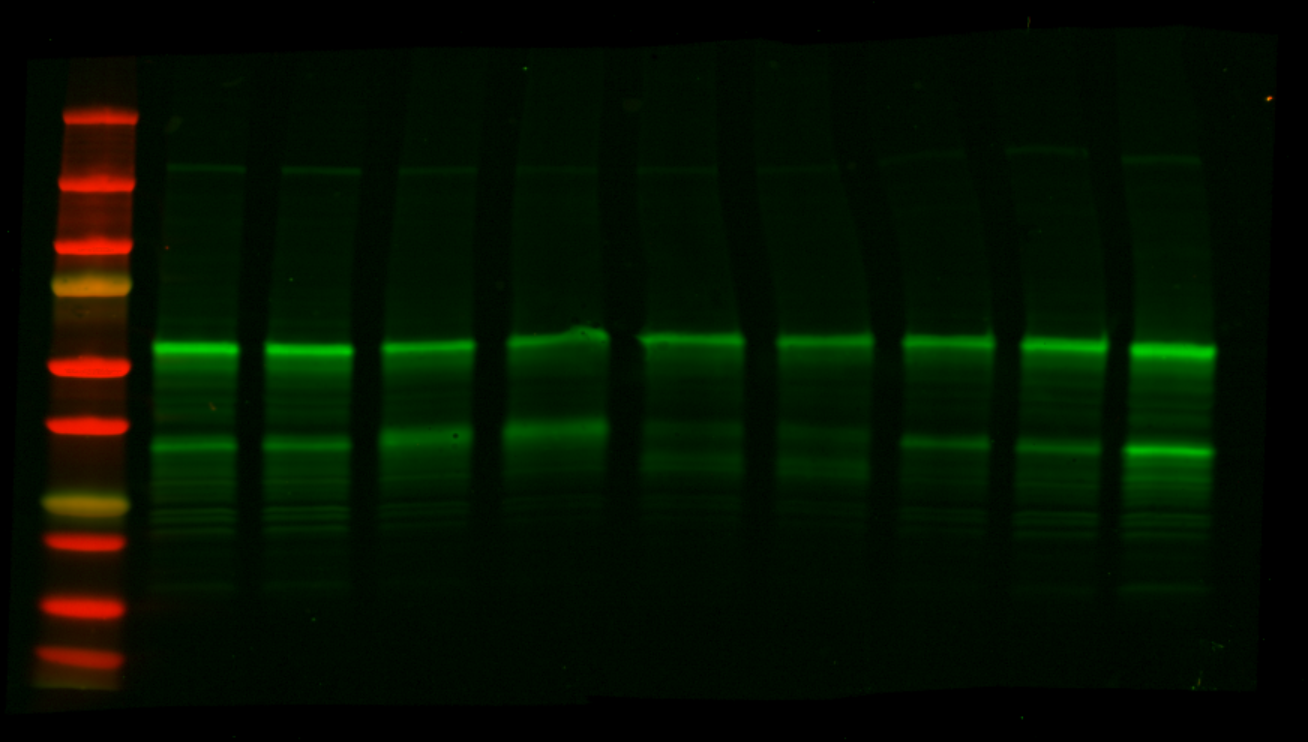
**

**GSDME-N**

**GSDME-FL**

MW 1 2 3 4 5 6 7 8 9


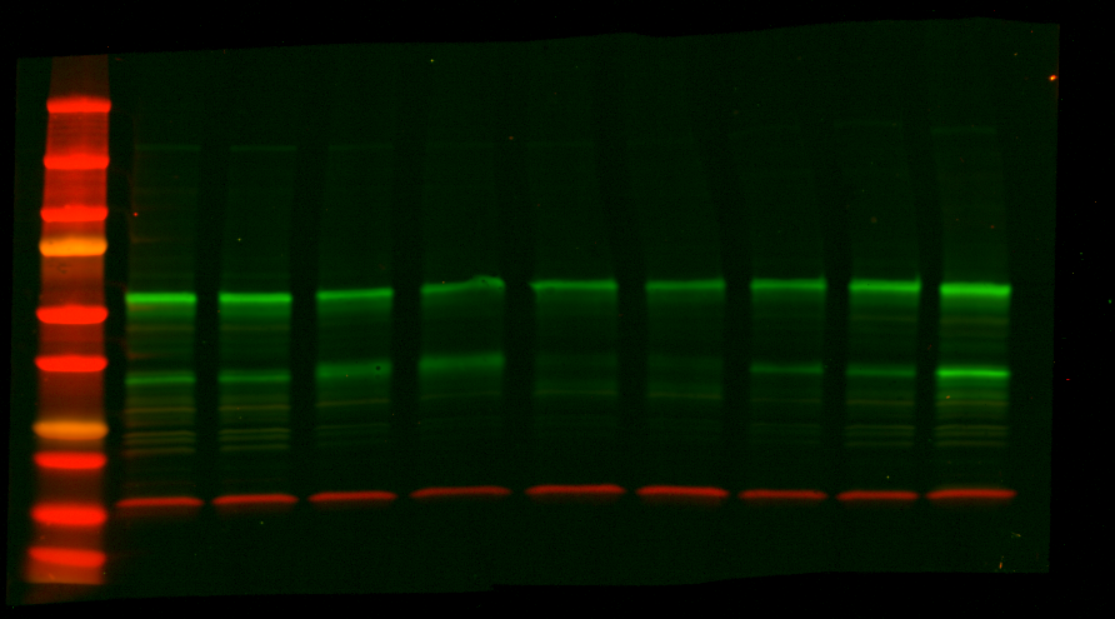


**GSDME-FL**

**GSDME-N**

**COX IV**

**MDA-MB231:** 1-2. Vehicle; 3-4. 0.5µM RSL3; 5-6. RSL3 + Fer1; 7-8. RSL3 + zVADfmk; 9. Sample not included in this study. MW = molecular weight (BioRad Precision Plus Protein Dual Color Standards, Cat. No. 1610374).

Top: GSDME-N signal acquisition. Bottom: COX IV loading control measured on the same Western blots, after GSDME-N signal detection, following additional washes (no stripping).

MW 1 2 3 4 5 6 7 8 9


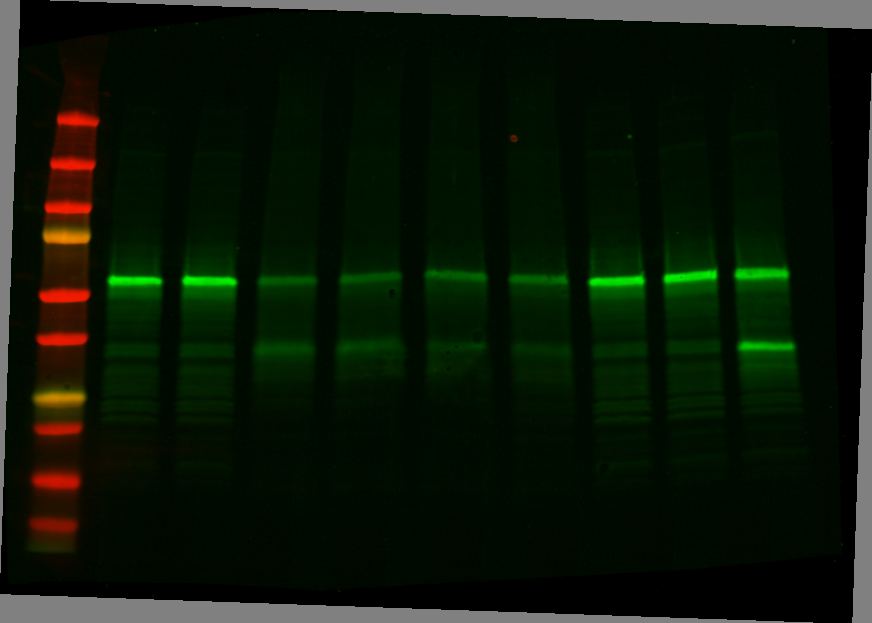


**GSDME-FL**

**GSDME-N**

MW 1 2 3 4 5 6 7 8 9


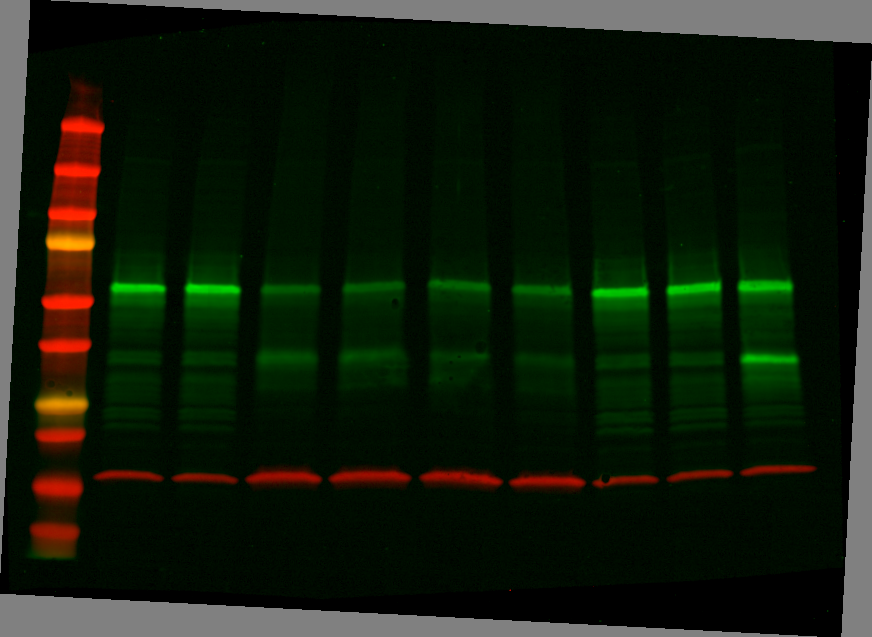


**GSDME-FL**

**GSDME-N**

**COX IV**

**Uncropped Western Blot Images for Supplemental Figure S5:**

1–3. Samples not included in this study; 4. THP1, vehicle; 5. THP1, LPS + 20 µM nigericin; 6. HAP1, GSDMD knock out. MW = molecular weight (LICORBio, Chameleon® Duo Pre-stained Protein Ladder, Cat. No. 928-60000).

MW 1 2 3 4 5 6 MW MW


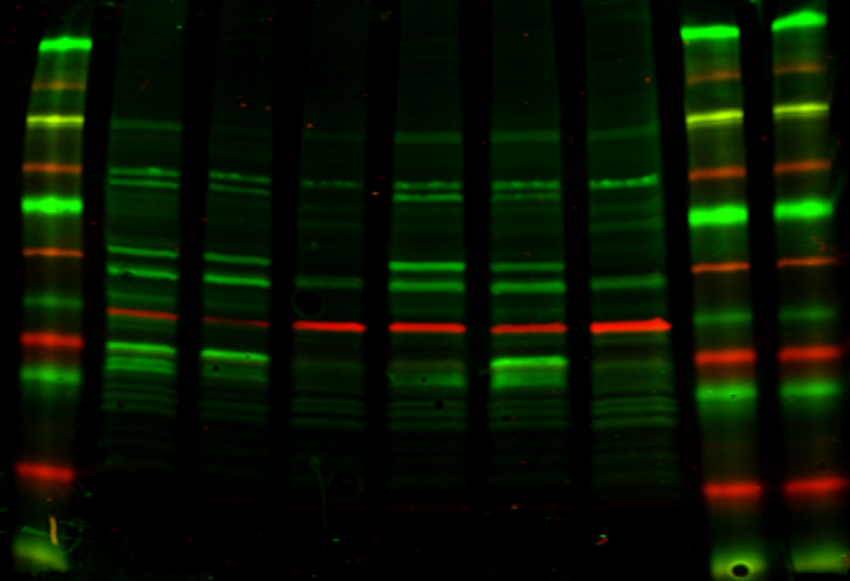


**GSDMD-N**

**GAPDH**

**GSDMD-FL**

**Uncropped Western Blot Images for Supplemental Figure S7:**

(Supplemental Figure S7, A) 1. NCI-H522, vehicle; 2. NCI-H522, RSL3; 3. ACHN, vehicle; 4. ACHN, RSL3; 5. UO-31, vehicle; 6. UO-31, RSL3; 7. SU-DHL-5, vehicle; 8. SU-DHL-5, RSL3; 9. BxPC3, vehicle. (Supplemental Figure S7, B) 11. NCI-H522, vehicle; 12. NCI-H522, RSL3; 13. ACHN, vehicle; 14. ACHN, RSL3; 15. UO-31, vehicle; 16. UO-31, RSL3; 17. SU-DHL-5, vehicle; 18. SU-DHL-5, RSL3; 19. BxPC3, vehicle.

MW = molecular weight (BioRad Precision Plus Protein Dual Color Standards, #1610374).

Top: STING and cGAS signal acquisition. Bottom: COX IV loading control measured on the same Western blots, after STING and cGAS signal detection, following additional washes (no stripping).

MW 1 2 3 4 5 6 7 8 9 MW 11 12 13 14 15 16 17 18 19


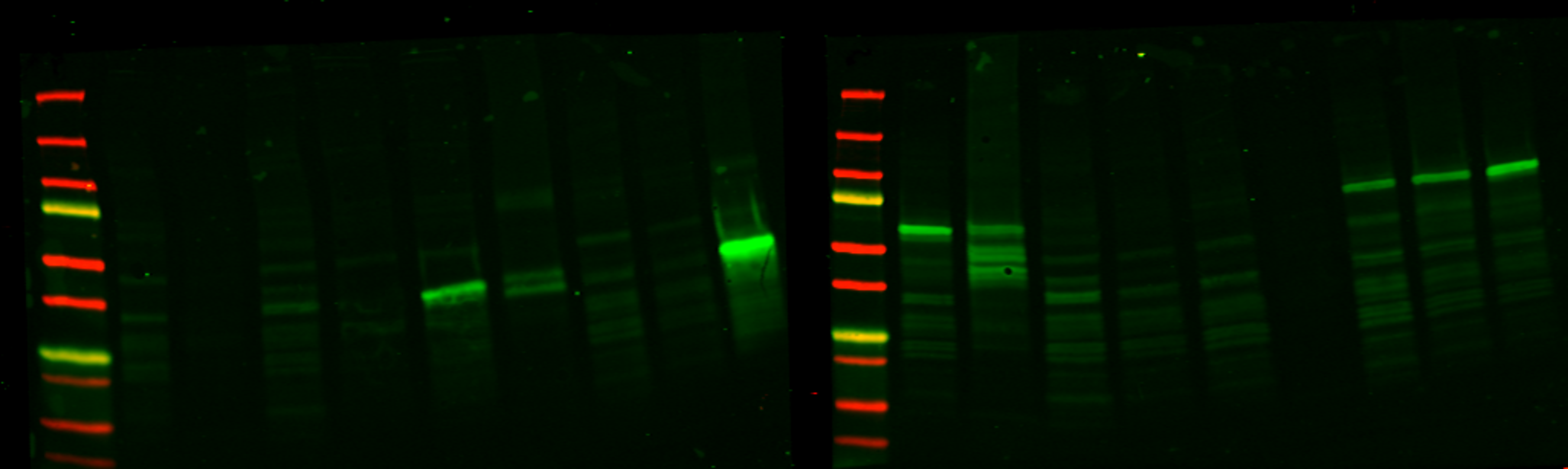


**cGAS**

**STING1**

MW 1 2 3 4 5 6 7 8 9 MW 11 12 13 14 15 16 17 18 19


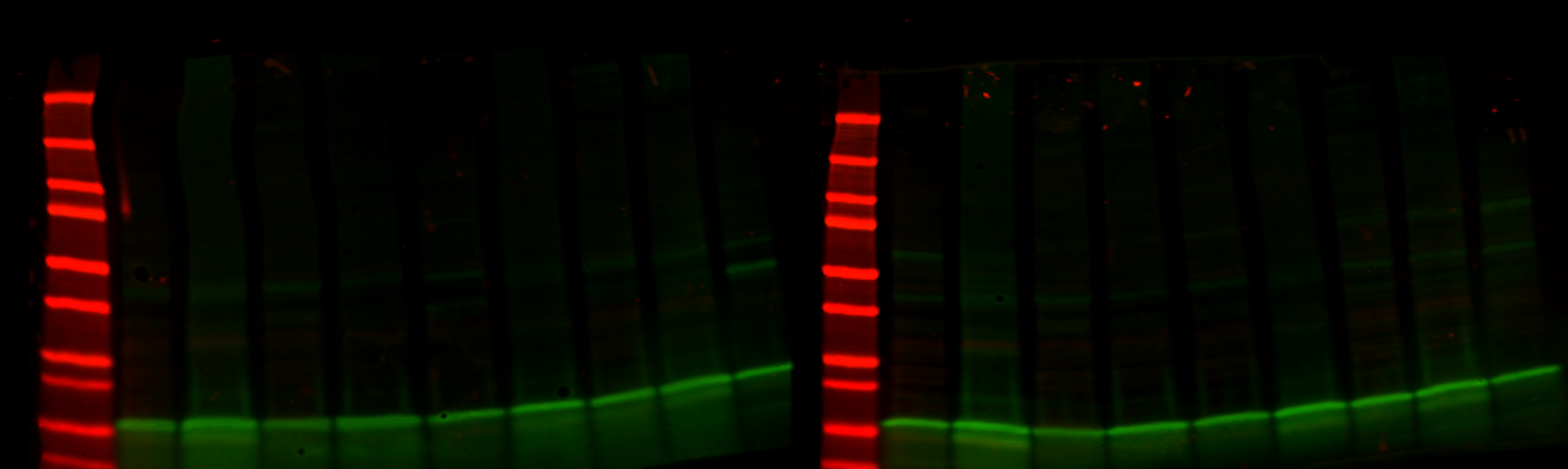


**COX IV**

**Uncropped Western Blot Images for Supplemental Figure S9:**

Top (pMLKL): Left Blot: 1. NCI-H522, vehicle; 2. NCI-H522, RSL3; 3. NCI-H522, RSL3 + Nec1; 4. ACHN, vehicle; 5. ACHN, RSL3; 6. ACHN, RSL3 + Nec1; 7. HT-29, vehicle; 8. HT-29, Tolinapant + TNFα + Emricasan. Right Blot: 9. SU-DHL-5, vehicle; 10. SU-DHL-5, RSL3; 11. SU-DHL-5, RSL3 + Nec1. MW = molecular weight (BioRad Precision Plus Protein Dual Color Standards, #1610374).

Bottom (total MLKL): the blot measuring pMLKL was stripped (LICOR, NewBlot™ Nitrocellulose 5X Stripping Buffer Cat. No. 929-97301) and reprobed for total MLKL.

MW 1 2 3 4 5 6 7 8 MW MW 9 10 11 MW
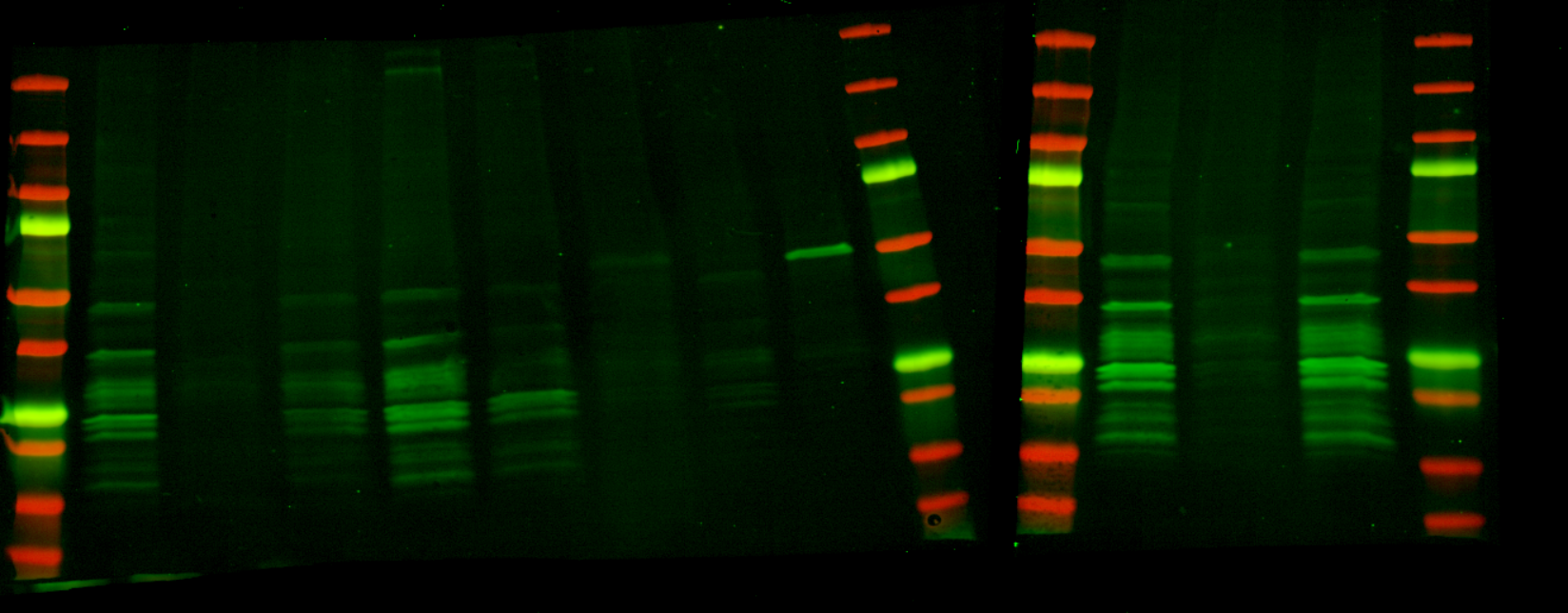


**pMLKL**

Bottom (total MLKL):

MW 1 2 3 4 5 6 7 8 MW MW 9 10 11 MW


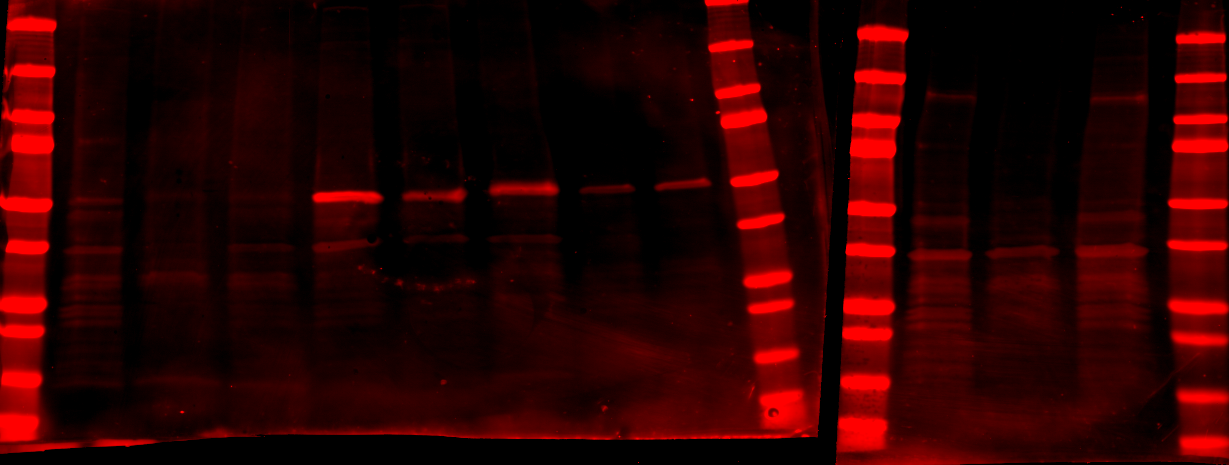


**Total MLKL**

# Supplemental references:

1. Aglietti RA, Estevez A, Gupta A, Ramirez MG, Liu PS, Kayagaki N, et al. GsdmD p30 elicited by caspase-11 during pyroptosis forms pores in membranes. Proc Natl Acad Sci U S A. 2016;113(28):7858-63.

2. Herrick WG, Govindharajulu J, Parchment RE, Doroshow JH, Srivastava AK. Abstract 6143: Novel multiplexed assays of ferroptosis, pyroptosis and necroptosis biomarkers for translational studies. Cancer Research. 2023;83(7_Supplement):6143-.
